# Supplementary figures and images for: Silencing of SRRM4 suppresses microexon inclusion and promotes tumor growth across cancers
Source: PLoS Biol. 2021 Feb 23;19(2):e3001138. doi: 10.1371/journal.pbio.3001138 (PMC7935315; doi:10.1371/journal.pbio.3001138)

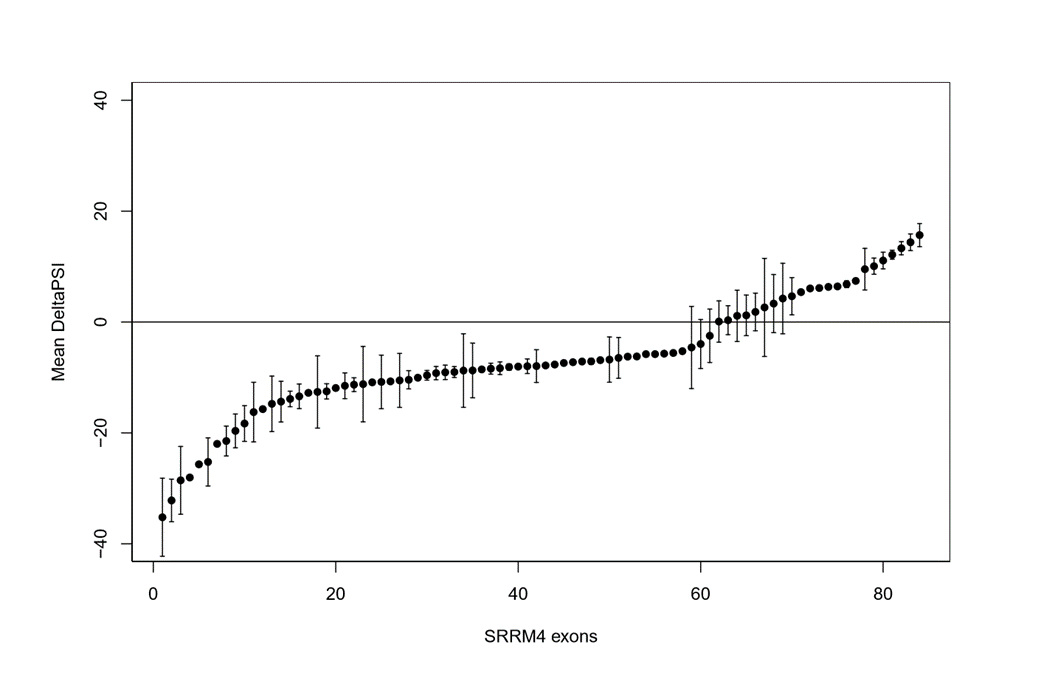

Supplement: S1 Fig — Error bars represent the standard error of ΔPSI across the 9 cancer types. Only significantly changing exons (q < 0.01) with ΔPSI ≥ 5 are shown. Data underlying this figure can be found in S1 Data figures. PSI, percent spliced in; SRRM4, Serine/Arginine Repetitive Matrix 4. (TIF) [file pbio.3001138.s001.tif]

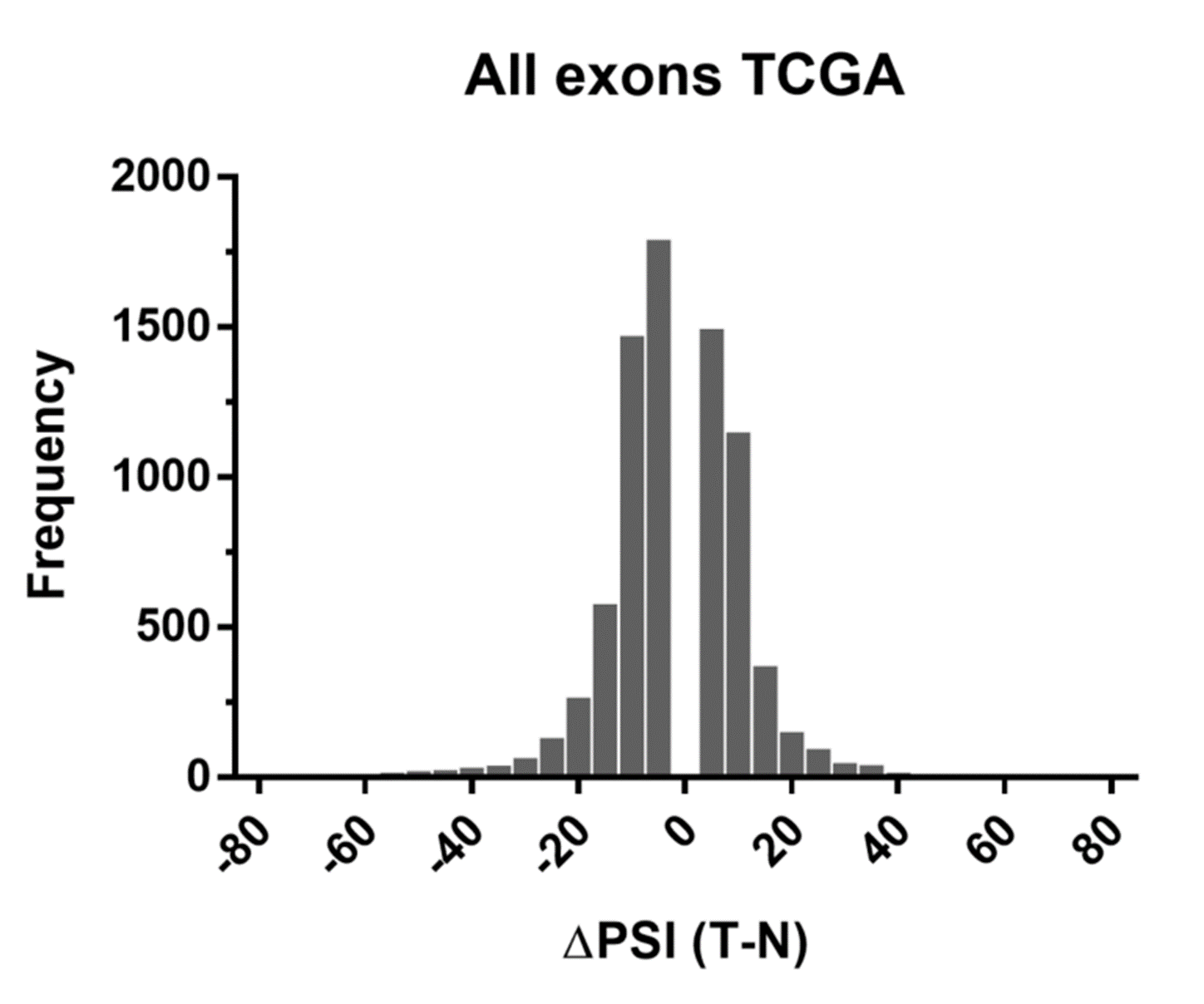

Supplement: S2 Fig — Only significantly changing exons (q < 0.01) with ΔPSI ≥ 5 are shown. Data underlying this figure can be found in S3 Data. PSI, percent spliced in; TCGA, The Cancer Genome Atlas. (TIF) [file pbio.3001138.s002.tif]

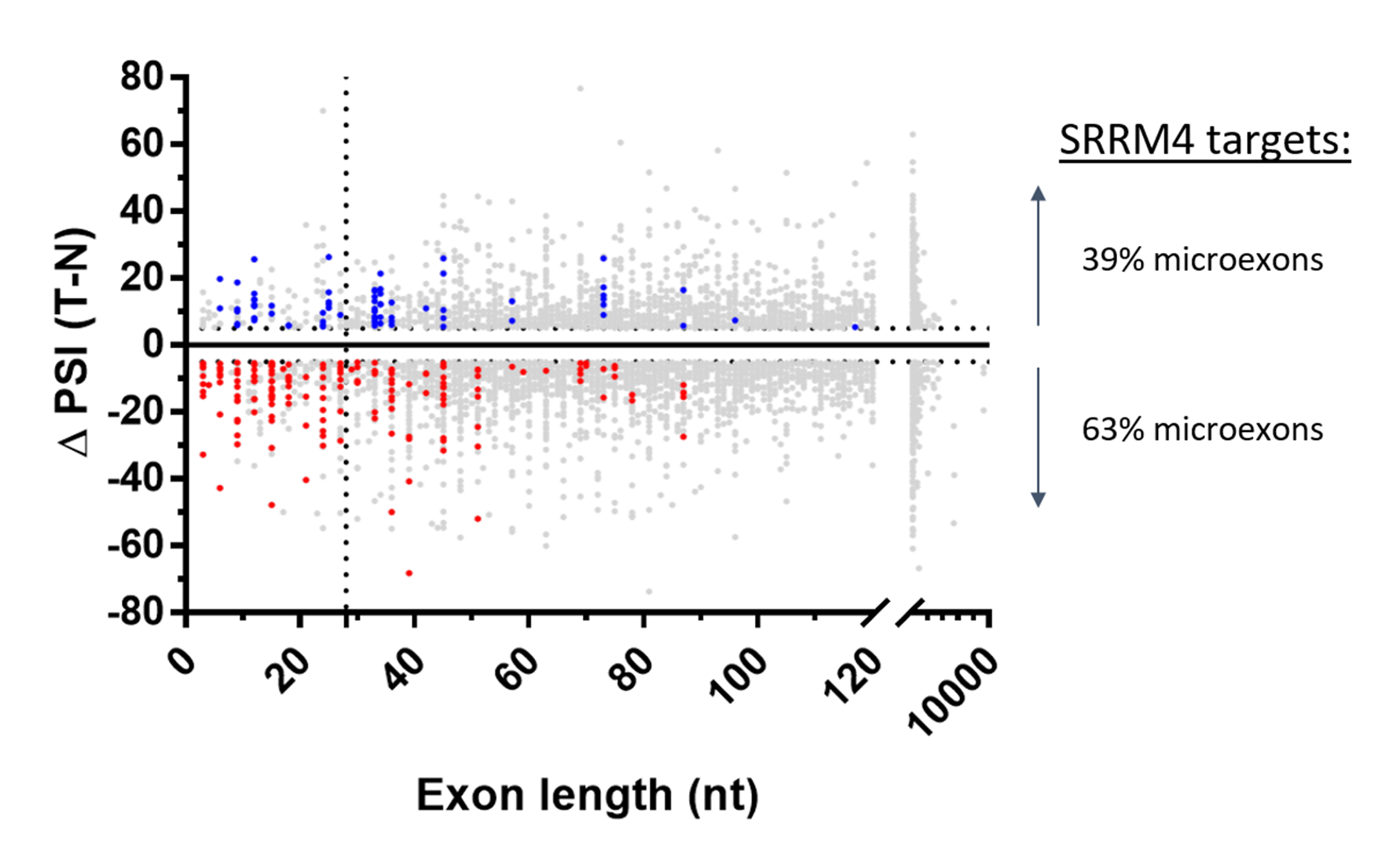

Supplement: S3 Fig — Blue points are SRRM4 target exons increased in tumors (T) vs. normal tissue (N); red are SRRM4 target exons decreased in tumors; gray are non-SRRM4 targets. Only significantly changing exons (q < 0.01) with ΔPSI ≥ 5 are shown. Vertical dotted line separates microexons (≤27 nt) from non-microexons (>27 nt). Data underlying this figure can be found in S3 Data. nt, nucleotide; PSI, percent spliced in; SRRM4, Serine/Arginine Repetitive Matrix 4. (TIF) [file pbio.3001138.s003.tif]

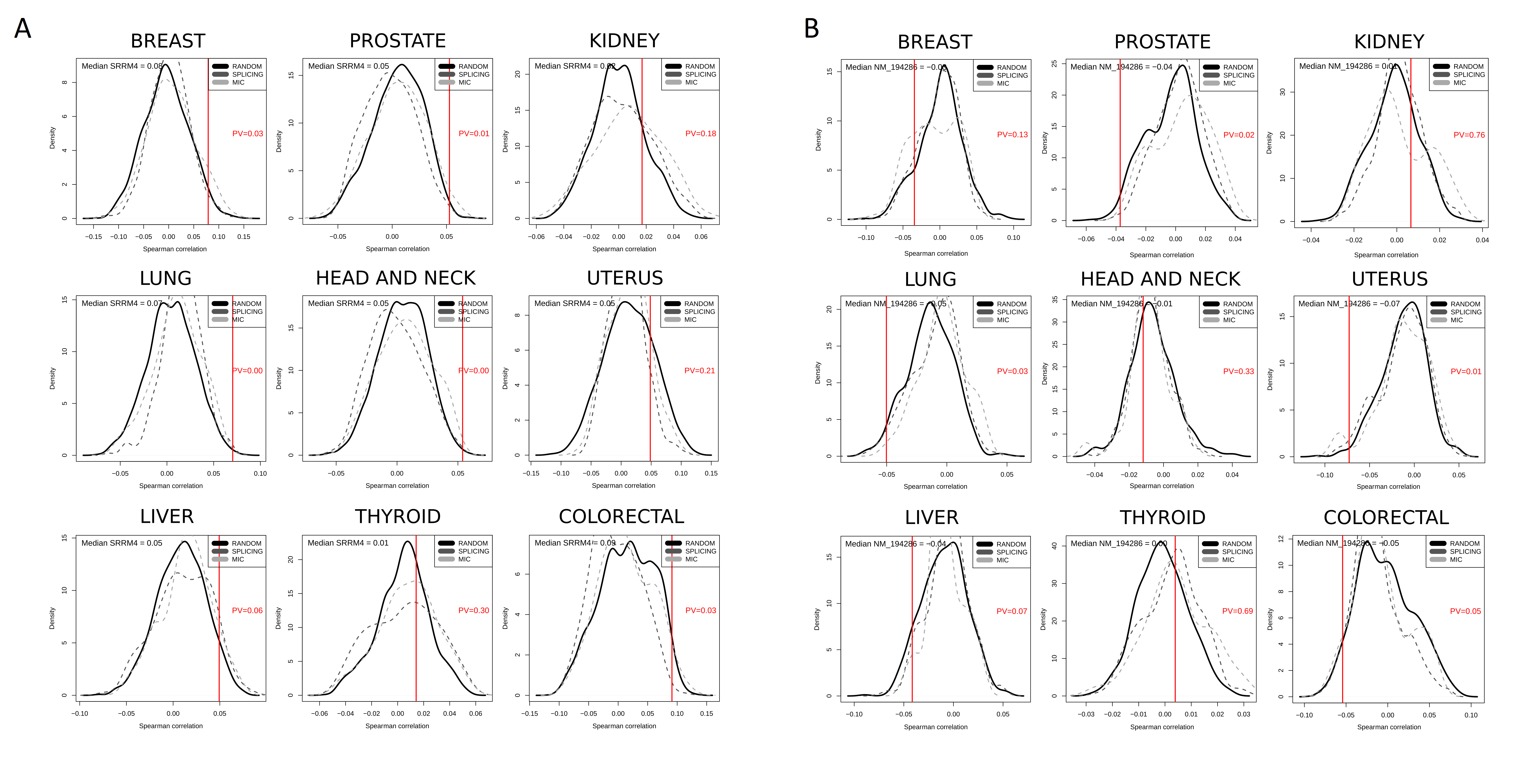

Supplement: S4 Fig — Median Spearman correlation of (A) SRRM4 expression and (B) SRRM4 methylation with SRRM4 target exons PSI across TCGA tumor samples. The significance of the correlation was determined based on a randomization test within a background of 5,000 random genes background, as well as 2 other backgrounds of 425 GO splicing–related genes and 233 microexon-related genes. Data underlying this figure can be found in S1 Data figures. GO, gene ontology; PSI, percent spliced in; SRRM4, Serine/Arginine Repetitive Matrix 4; TCGA, The Cancer Genome Atlas. (TIF) [file pbio.3001138.s004.tif]

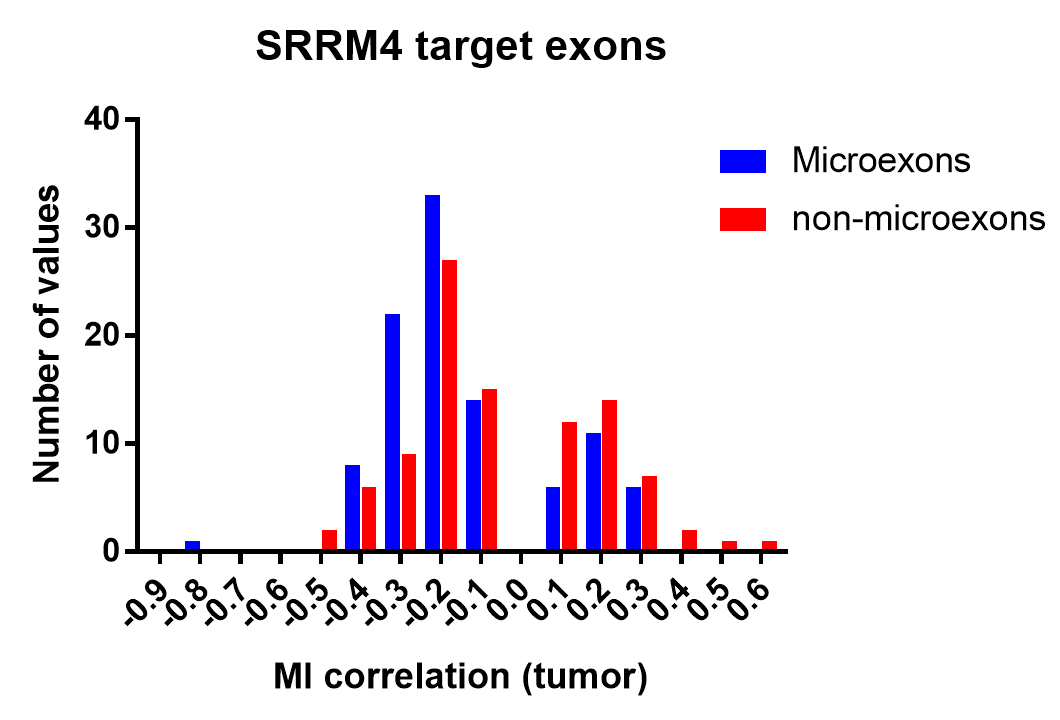

Supplement: S5 Fig — Only exons with |ΔPSI| > 5 and q < 0.01 are shown. Data underlying this figure can be found in S1 Data figures. MI, mitotic index; nt, nucleotide; PSI, percent spliced in. (TIF) [file pbio.3001138.s005.tif]

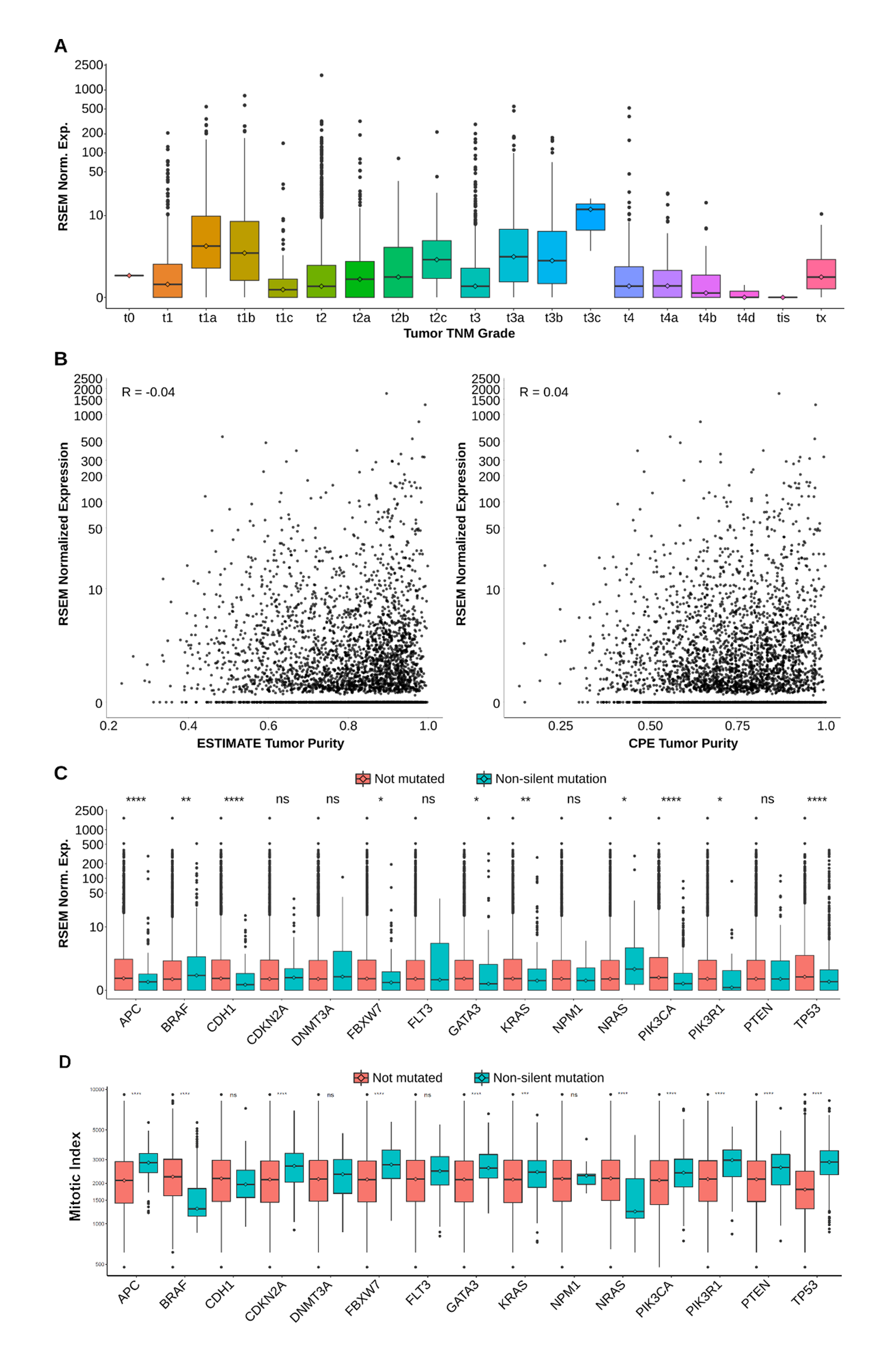

Supplement: S6 Fig — (A) SRRM4 expression across different TNM tumor stages. (B) Scatter plots and Spearman correlations between SRRM4 and 2 tumor purity estimates (ESTIMATE and CPE). (C) Association of SRRM4 expression with the presence or absence of mutations in cancer driver genes. Significance is determined by a 2-tailed unpaired Wilcoxon–Mann–Whitney test: *p < 0.05, ** p < 0.01, *** p < 0.001, **** p < 0.0001. Data underlying this figure can be found in S1 Data figures. SRRM4, Serine/Arginine Repetitive Matrix 4; TCGA, The Cancer Genome Atlas; TNM, tumor/node/metastasis. (TIF) [file pbio.3001138.s006.tif]

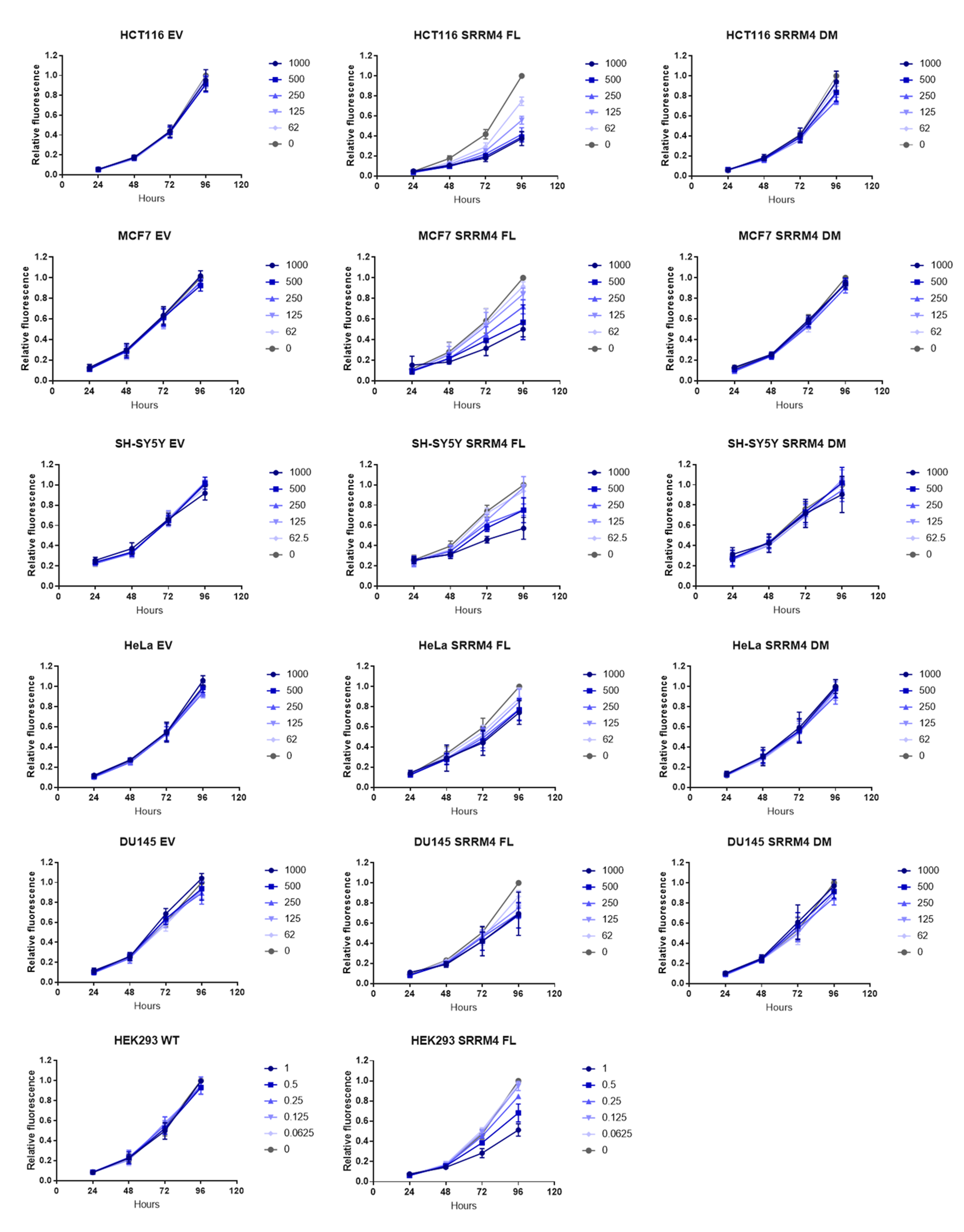

Supplement: S7 Fig — Cells were transduced with EV, WT SRRM4, or DM SRRM4 and treated with indicated concentrations of doxycycline (ng/mL). All lines were generated by lentiviral transduction (see Methods) except for HEK293, which was generated by a Flp-In system [22]. Results shown are averages of 2–3 independent experiments, and error bars indicate SEM. Data underlying this figure can be found in S1 Data figures. DM, deletion mutant; EV, empty vector; SEM, standard error of the mean; SRRM4, Serine/Arginine Repetitive Matrix 4; WT, wild-type. (TIF) [file pbio.3001138.s007.tif]

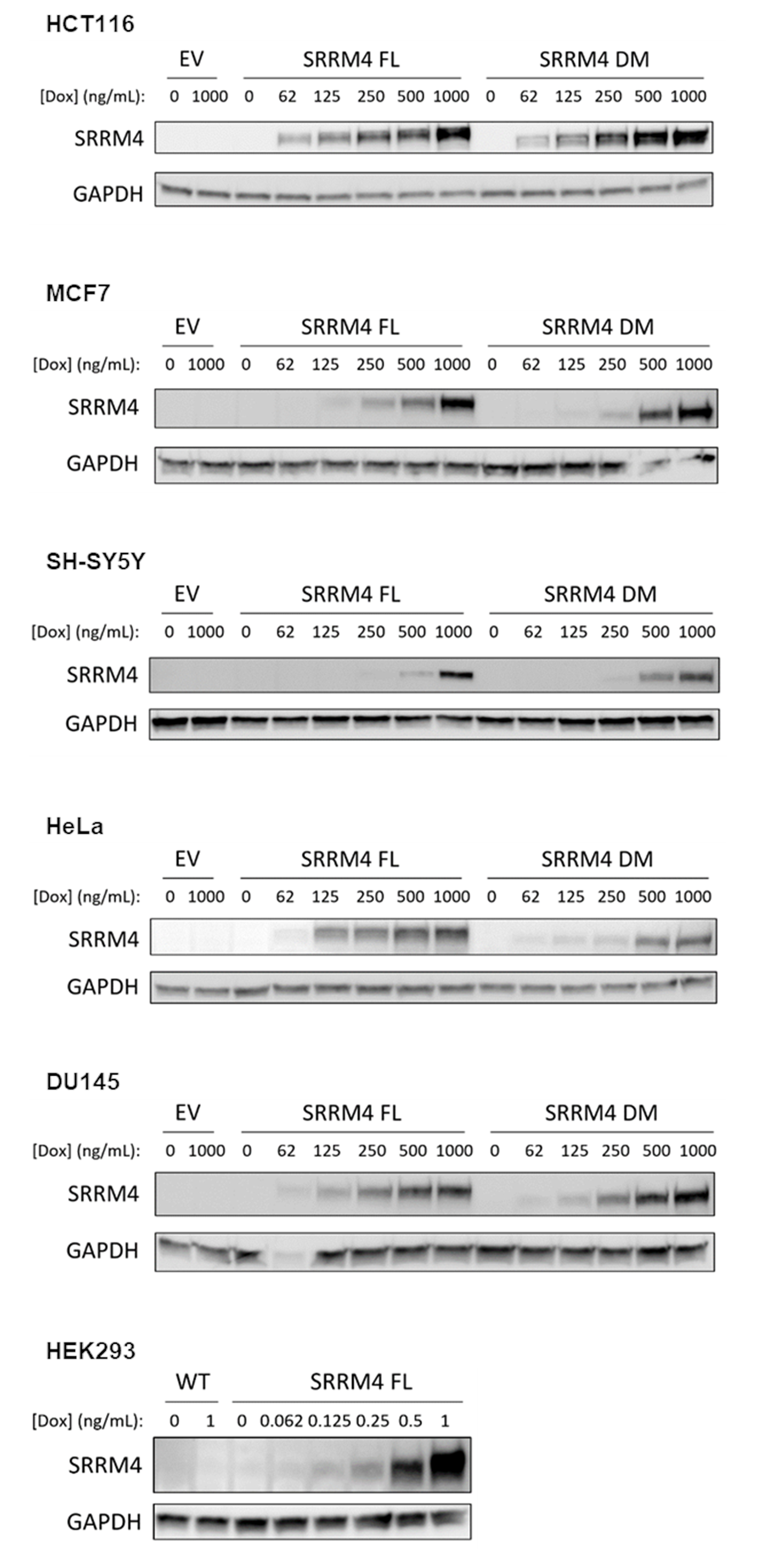

Supplement: S8 Fig — Cells were treated for 24 h with the indicated concentrations of doxycycline. Results shown are representative of 2 independent experiments. DM, deletion mutant; SRRM4, Serine/Arginine Repetitive Matrix 4; WT, wild-type. (TIF) [file pbio.3001138.s008.tif]

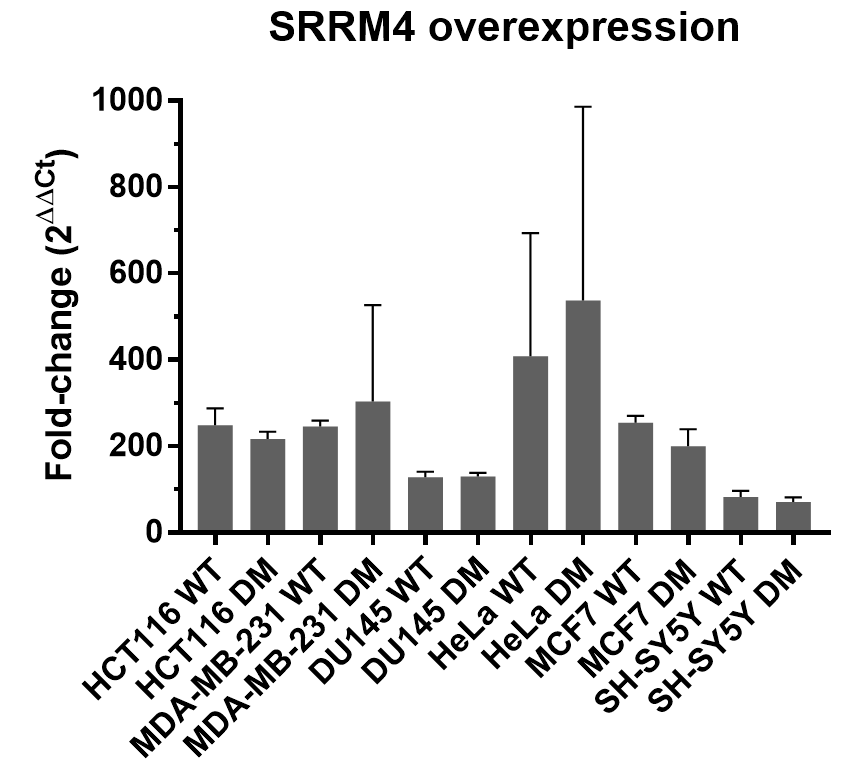

Supplement: S9 Fig — SRRM4 overexpression relative to uninduced cells was quantified by qPCR using the ΔΔCt method, with GAPDH as internal control. Error bars represent SEM of 2–3 independent experiments. Data underlying this figure can be found in S1 Data figures. Ct, cycle threshold; qPCR, quantitative PCR; SEM, standard error of the mean; SRRM4, Serine/Arginine Repetitive Matrix 4. (TIF) [file pbio.3001138.s009.tif]

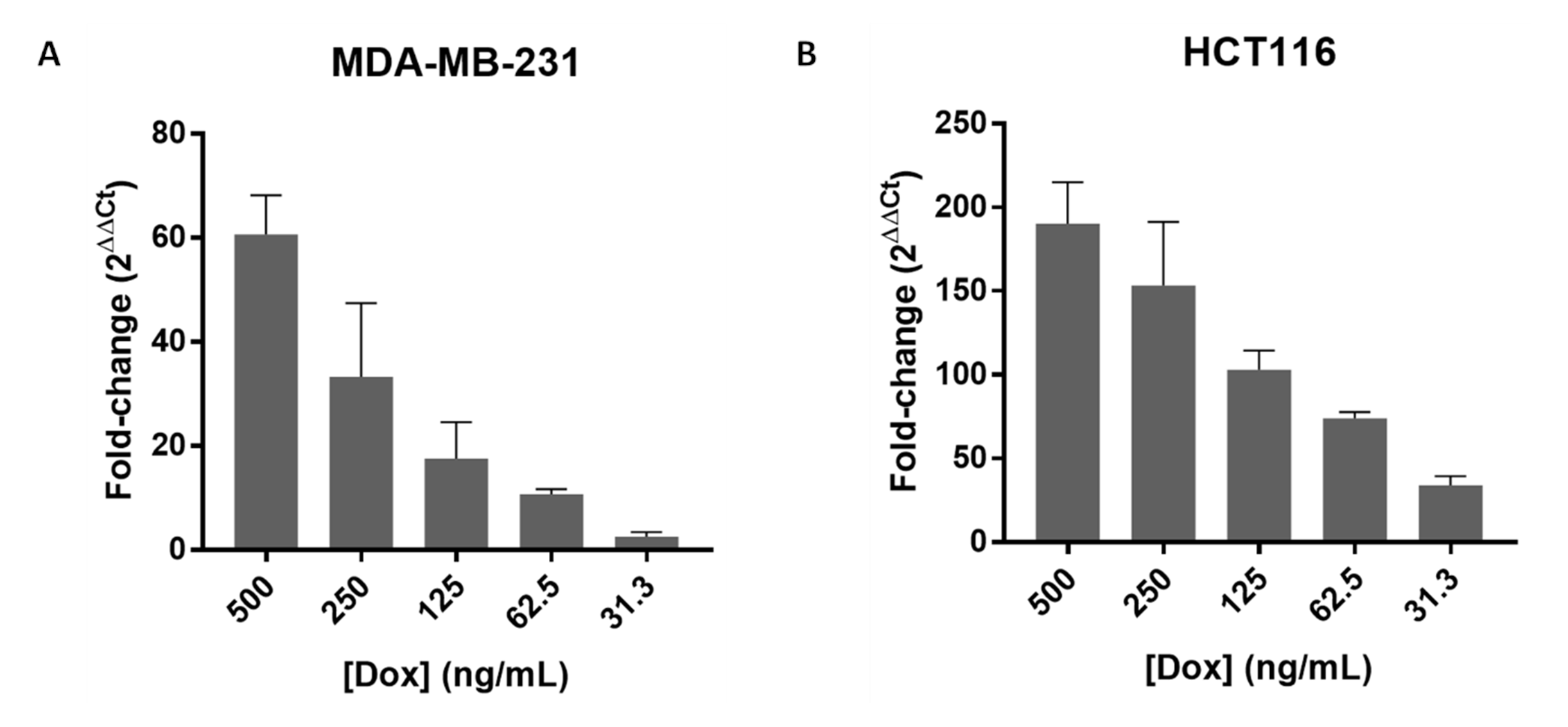

Supplement: S10 Fig — Expression levels of SRRM4 in (A) MDA-MB-231 cells and (B) HCT116 cells after 24 h induction with indicated concentrations of doxycycline. SRRM4 overexpression relative to uninduced cells was quantified by qPCR using the ΔΔCt method, with GAPDH as internal control. Error bars represent standard deviation of 2 independent experiments. Data underlying this figure can be found in S1 Data figures. Ct, cycle threshold; qPCR, quantitative PCR; SRRM4, Serine/Arginine Repetitive Matrix 4. (TIF) [file pbio.3001138.s010.tif]

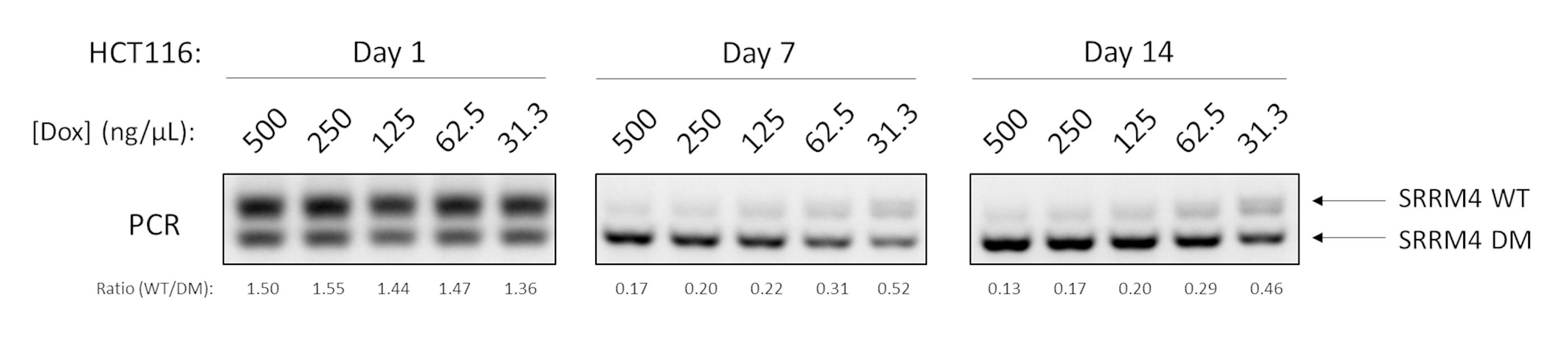

Supplement: S11 Fig — WT/DM ratios quantified by densitometry are shown below each sample. Results shown are representative of 2 experiments. DM, deletion mutant; WT, wild-type. (TIF) [file pbio.3001138.s011.tif]

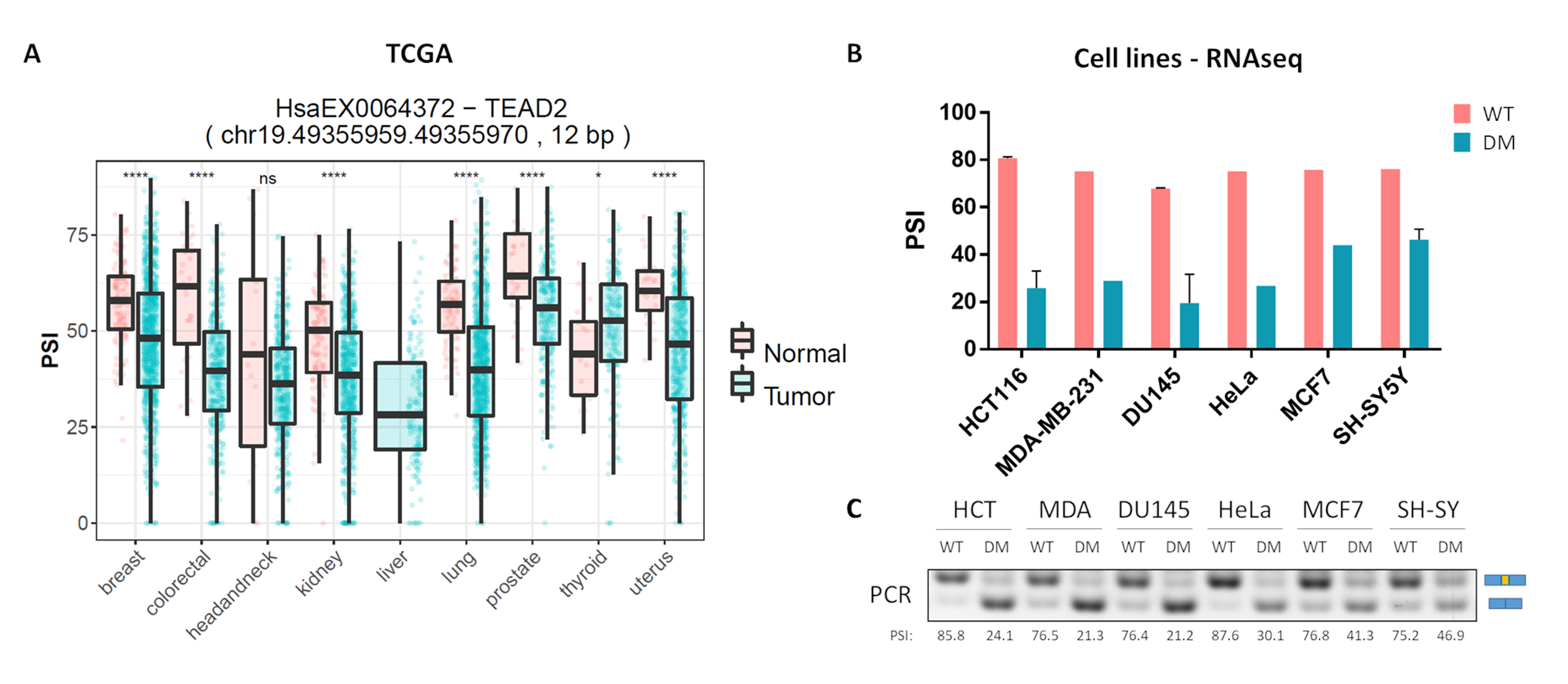

Supplement: S12 Fig — (A) The microexon (VastDB event ID: HsaEX0064372) displays significantly decreased inclusion in 6/9 cancer types (2-tailed unpaired Wilcoxon–Mann–Whitney test, * p < 0.05, ** p < 0.01, *** p < 0.001, **** p < 0.0001). No data are shown for normal liver due to a lack of sufficient read coverage in at least 20 samples. Genomic coordinates shown are from human genome reference build hg38. Data underlying this figure can be found in S1 Data figures. (B) The microexon displays increased inclusion in all 6 cancer cell lines after WT SRRM4 expression, relative to DM SRRM4, as assessed by RNA-seq. Data underlying this figure can be found in S9 Data. (C) PCR validation of the microexon inclusion changes in the 6 cancer cell lines. PSIs quantified by densitometry are shown below each sample. DM, deletion mutant; nt, nucleotide; PSI, percent spliced in; RNA-seq, RNA sequencing; SRRM4, Serine/Arginine Repetitive Matrix 4; WT, wild-type. (TIF) [file pbio.3001138.s012.tif]

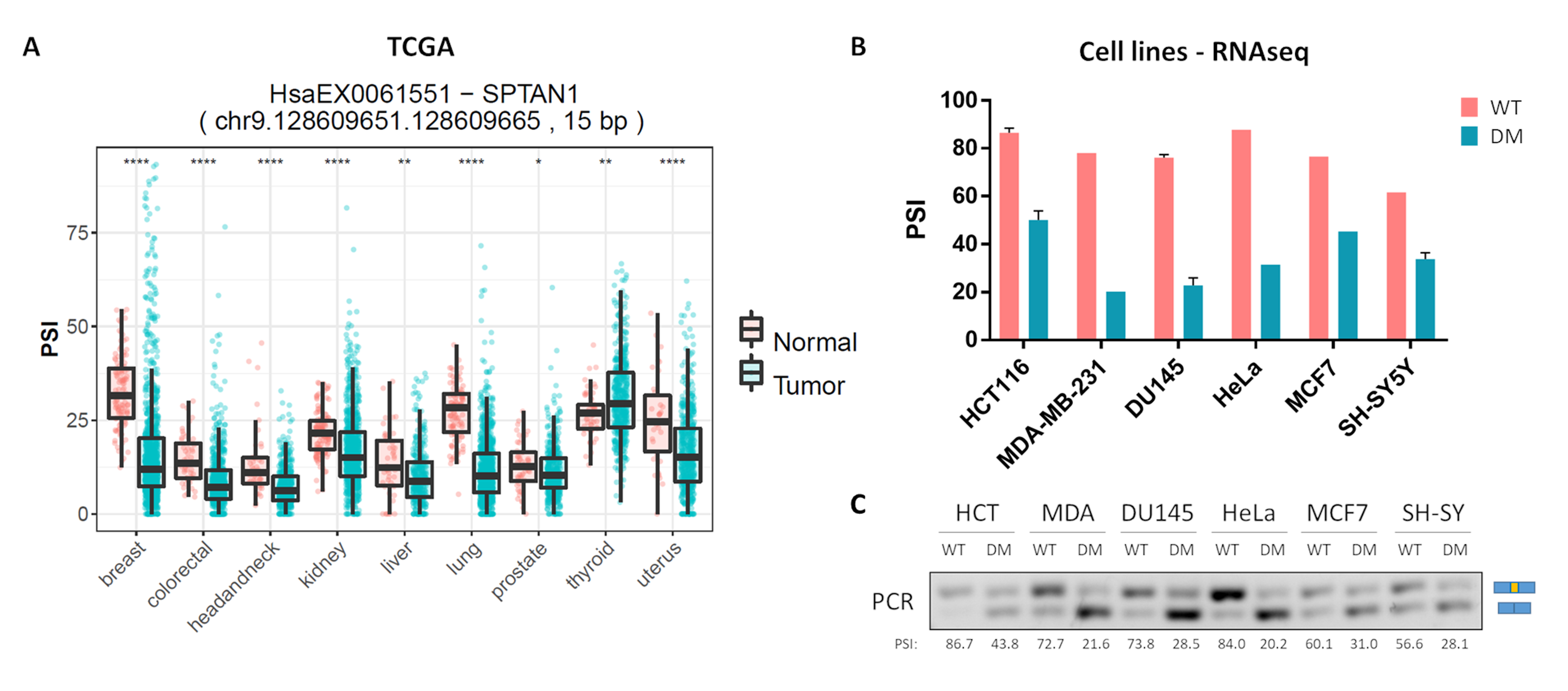

Supplement: S13 Fig — (A) The microexon (VastDB event ID: HsaEX0061551) displays significantly decreased inclusion in 8/9 cancer types (2-tailed unpaired Wilcoxon–Mann–Whitney test, * p < 0.05, ** p < 0.01, *** p < 0.001, **** p < 0.0001). Genomic coordinates shown are from human genome reference build hg38. Data underlying this figure can be found in S1 Data figures. (B) The microexon displays increased inclusion in all 6 cancer cell lines after WT SRRM4 expression, relative to DM SRRM4, as assessed by RNA-seq. Data underlying this figure can be found in S9 Data. (C) PCR validation of the microexon inclusion changes in the 6 cancer cell lines. PSIs quantified by densitometry are shown below each sample. DM, deletion mutant; nt, nucleotide; PSI, percent spliced in; RNA-seq, RNA sequencing; SRRM4, Serine/Arginine Repetitive Matrix 4; WT, wild-type. (TIF) [file pbio.3001138.s013.tif]

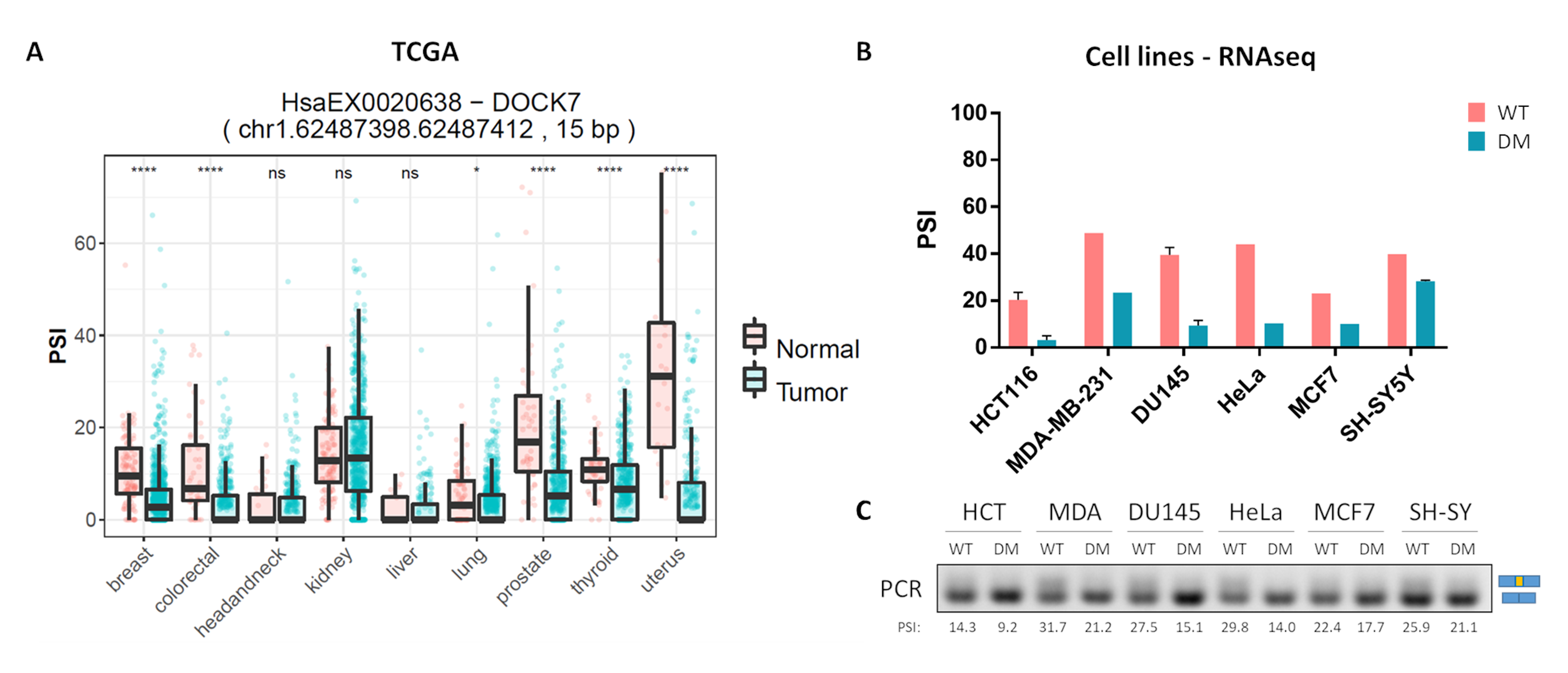

Supplement: S14 Fig — (A) The microexon (VastDB event ID: HsaEX0020638) displays significantly decreased inclusion in 6/9 cancer types (2-tailed unpaired Wilcoxon–Mann–Whitney test, * p < 0.05, ** p < 0.01, *** p < 0.001, **** p < 0.0001). Genomic coordinates shown are from human genome reference build hg38. Data underlying this figure can be found in S1 Data figures. (B) The microexon displays increased inclusion in all 6 cancer cell lines after WT SRRM4 expression, relative to DM SRRM4, as assessed by RNA-seq. Data underlying this figure can be found in S9 Data. (C) PCR validation of the microexon inclusion changes in the 6 cancer cell lines. PSIs quantified by densitometry are shown below each sample. DM, deletion mutant; nt, nucleotide; PSI, percent spliced in; RNA-seq, RNA sequencing; SRRM4, Serine/Arginine Repetitive Matrix 4; WT, wild-type. (TIF) [file pbio.3001138.s014.tif]

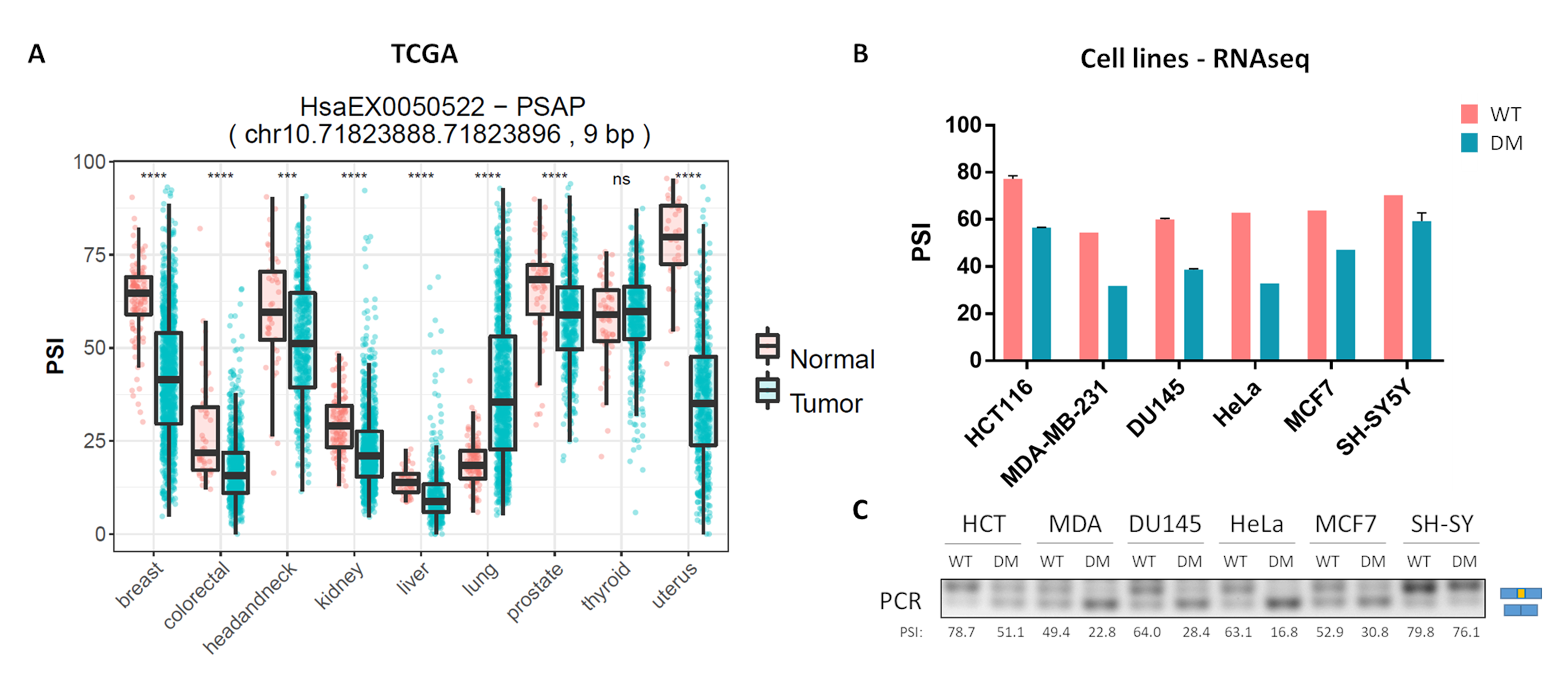

Supplement: S15 Fig — (A) The microexon (VastDB event ID: HsaEX0050522) displays significantly decreased inclusion in 7/9 cancer types (2-tailed unpaired Wilcoxon–Mann–Whitney test, * p < 0.05, ** p < 0.01, *** p < 0.001, **** p < 0.0001). Genomic coordinates shown are from human genome reference build hg38. Data underlying this figure can be found in S1 Data figures. (B) The microexon displays increased inclusion in all 6 cancer cell lines after WT SRRM4 expression, relative to DM SRRM4, as assessed by RNA-seq. Data underlying this figure can be found in S9 Data. (C) PCR validation of the microexon inclusion changes in the 6 cancer cell lines. PSIs quantified by densitometry are shown below each sample. DM, deletion mutant; nt, nucleotide; PSI, percent spliced in; RNA-seq, RNA sequencing; SRRM4, Serine/Arginine Repetitive Matrix 4; WT, wild-type. (TIF) [file pbio.3001138.s015.tif]

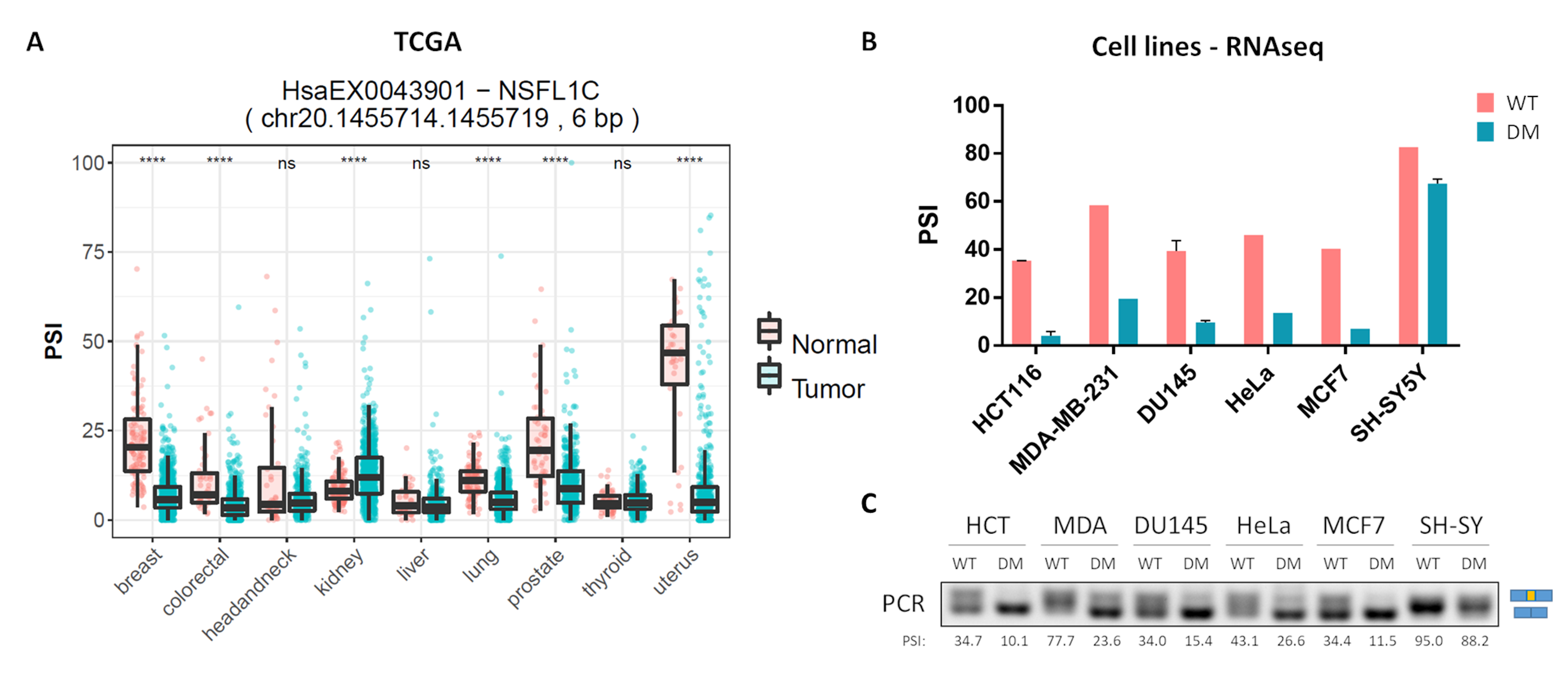

Supplement: S16 Fig — (A) The microexon (VastDB event ID: HsaEX0043901) displays significantly decreased inclusion in 5/9 cancer types (2-tailed unpaired Wilcoxon–Mann–Whitney test, * p < 0.05, ** p < 0.01, *** p < 0.001, **** p < 0.0001). Genomic coordinates shown are from human genome reference build hg38. Data underlying this figure can be found in S1 Data figures. (B) The microexon displays increased inclusion in all 6 cancer cell lines after WT SRRM4 expression, relative to DM SRRM4, as assessed by RNA-seq. Data underlying this figure can be found in S9 Data. (C) PCR validation of the microexon inclusion changes in the 6 cancer cell lines. PSIs quantified by densitometry are shown below each sample. DM, deletion mutant; nt, nucleotide; PSI, percent spliced in; RNA-seq, RNA sequencing; SRRM4, Serine/Arginine Repetitive Matrix 4; WT, wild-type. (TIF) [file pbio.3001138.s016.tif]

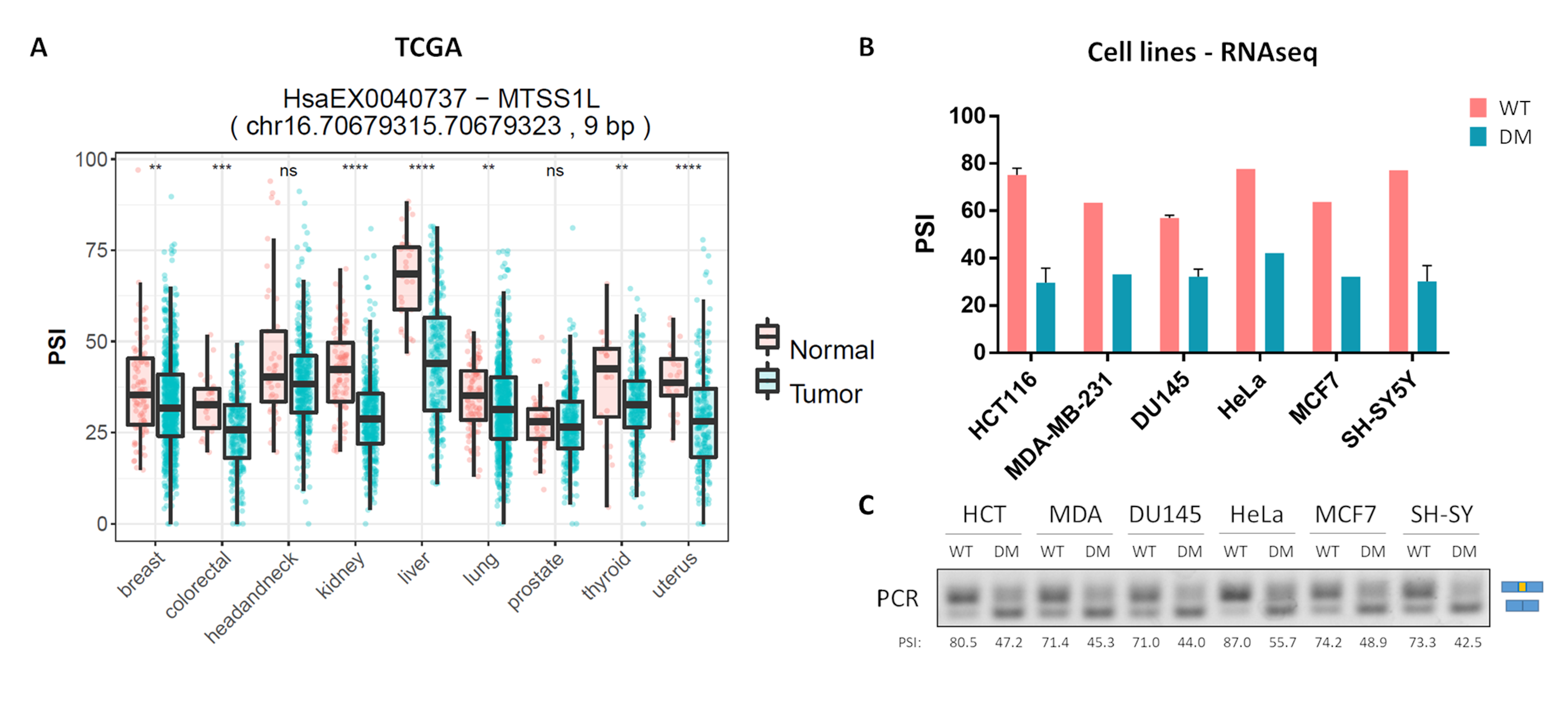

Supplement: S17 Fig — (A) The microexon (VastDB event ID: HsaEX0040737) displays significantly decreased inclusion in 7/9 cancer types (2-tailed unpaired Wilcoxon–Mann–Whitney test, * p < 0.05, ** p < 0.01, *** p < 0.001, **** p < 0.0001). Genomic coordinates shown are from human genome reference build hg38. Data underlying this figure can be found in S1 Data figures. (B) The microexon displays increased inclusion in all 6 cancer cell lines after WT SRRM4 expression, relative to DM SRRM4, as assessed by RNA-seq. Data underlying this figure can be found in S9 Data. (C) PCR validation of the microexon inclusion changes in the 6 cancer cell lines. PSIs quantified by densitometry are shown below each sample. DM, deletion mutant; nt, nucleotide; PSI, percent spliced in; RNA-seq, RNA sequencing; SRRM4, Serine/Arginine Repetitive Matrix 4; WT, wild-type. (TIF) [file pbio.3001138.s017.tif]
