# Supplementary material for: Silencing of SRRM4 suppresses microexon inclusion and promotes tumor growth across cancers
Source: PLoS Biol. 2021 Feb 23;19(2):e3001138. doi: 10.1371/journal.pbio.3001138 (PMC7935315; doi:10.1371/journal.pbio.3001138)
Supplement: S1 Raw Images — (PDF) [file pbio.3001138.s035.pdf]

Fig 5d

IB: SRRM4
(Chemiluminescence
LAS3000)

IB: GAPDH
(Chemiluminescence
LAS3000)

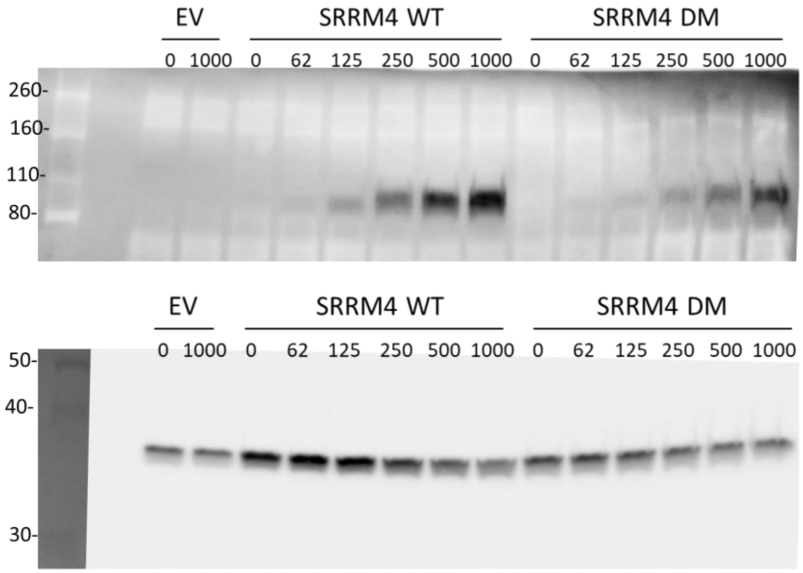

Fig 5f

PCR
(UV GelDoc)

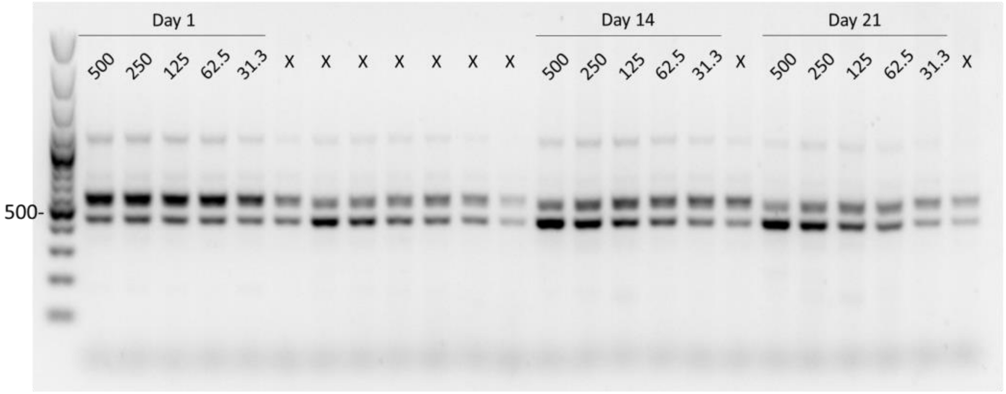

S10 Fig

PCR
(UV GelDoc)

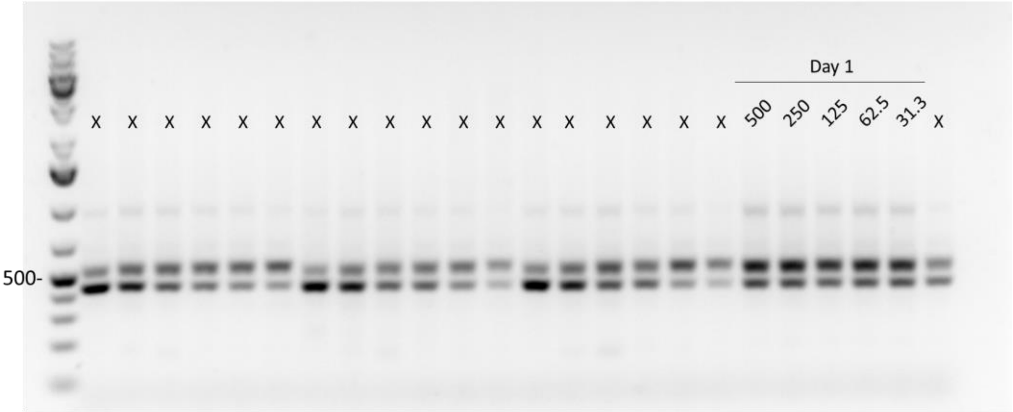

S10 Fig (2)

PCR
(UV GelDoc)

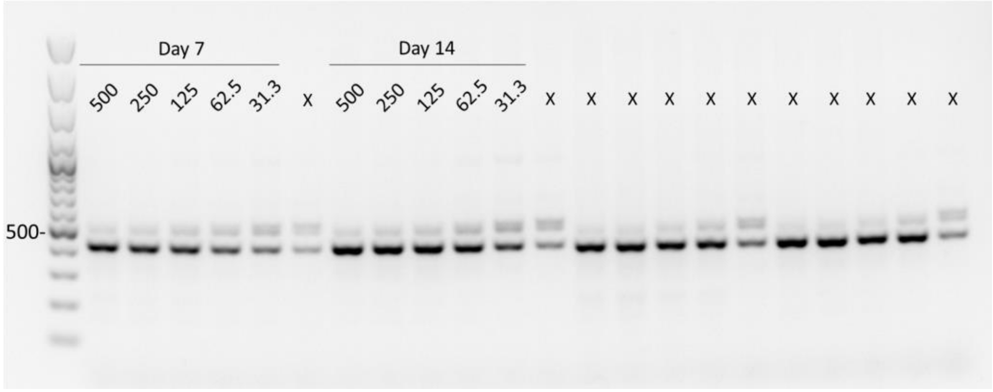

S11c Fig

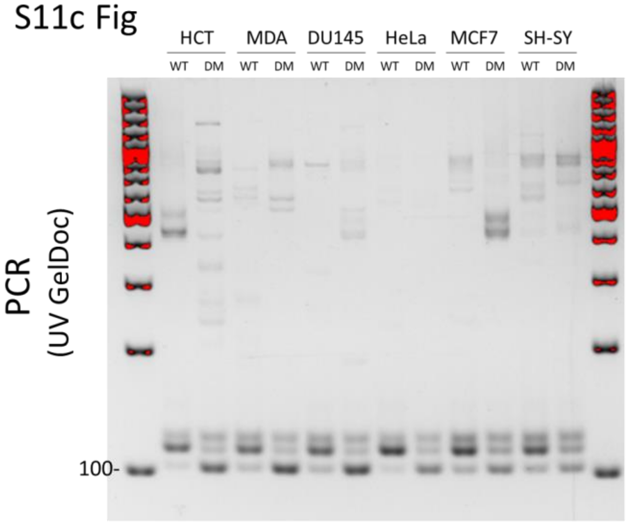

S14c Fig

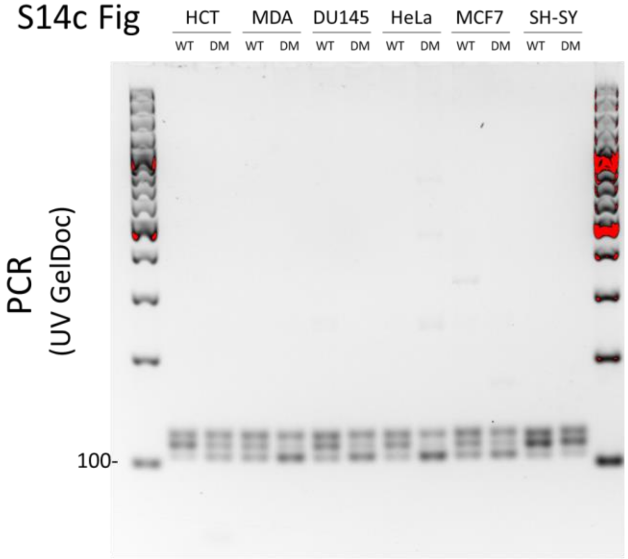

S12c Fig

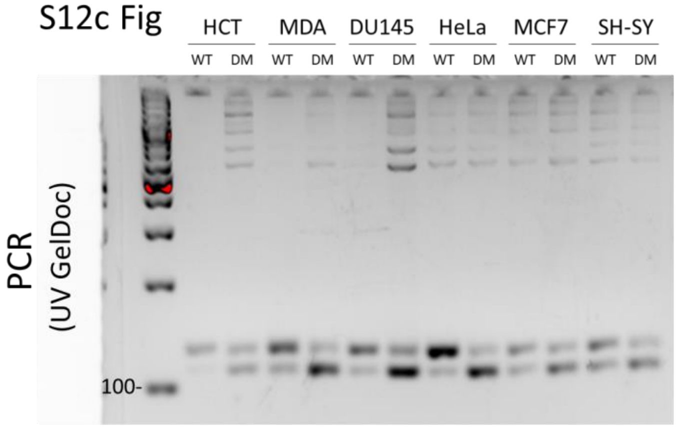

S15c Fig

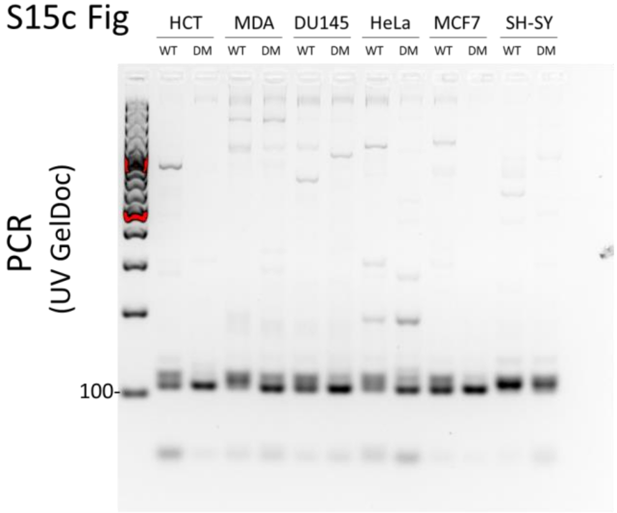

S13c Fig

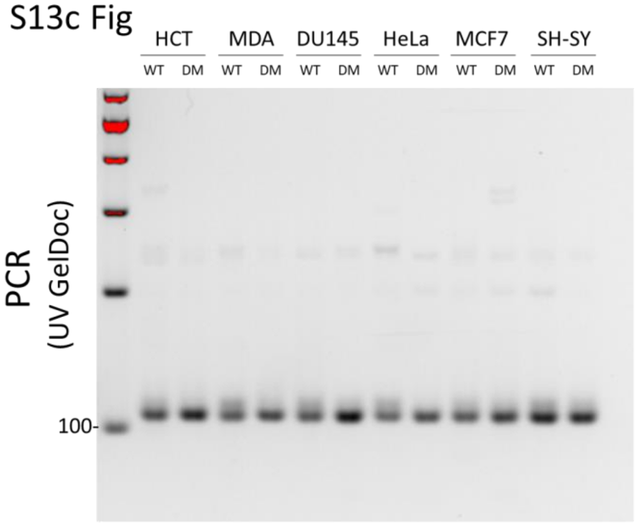

S16c Fig

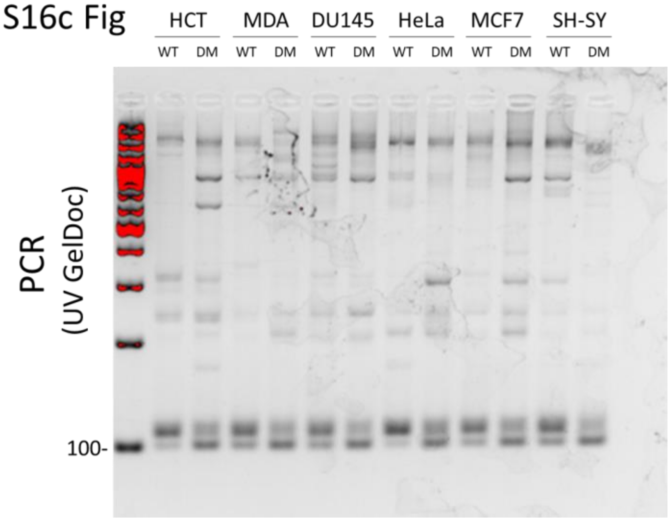

S7 Fig

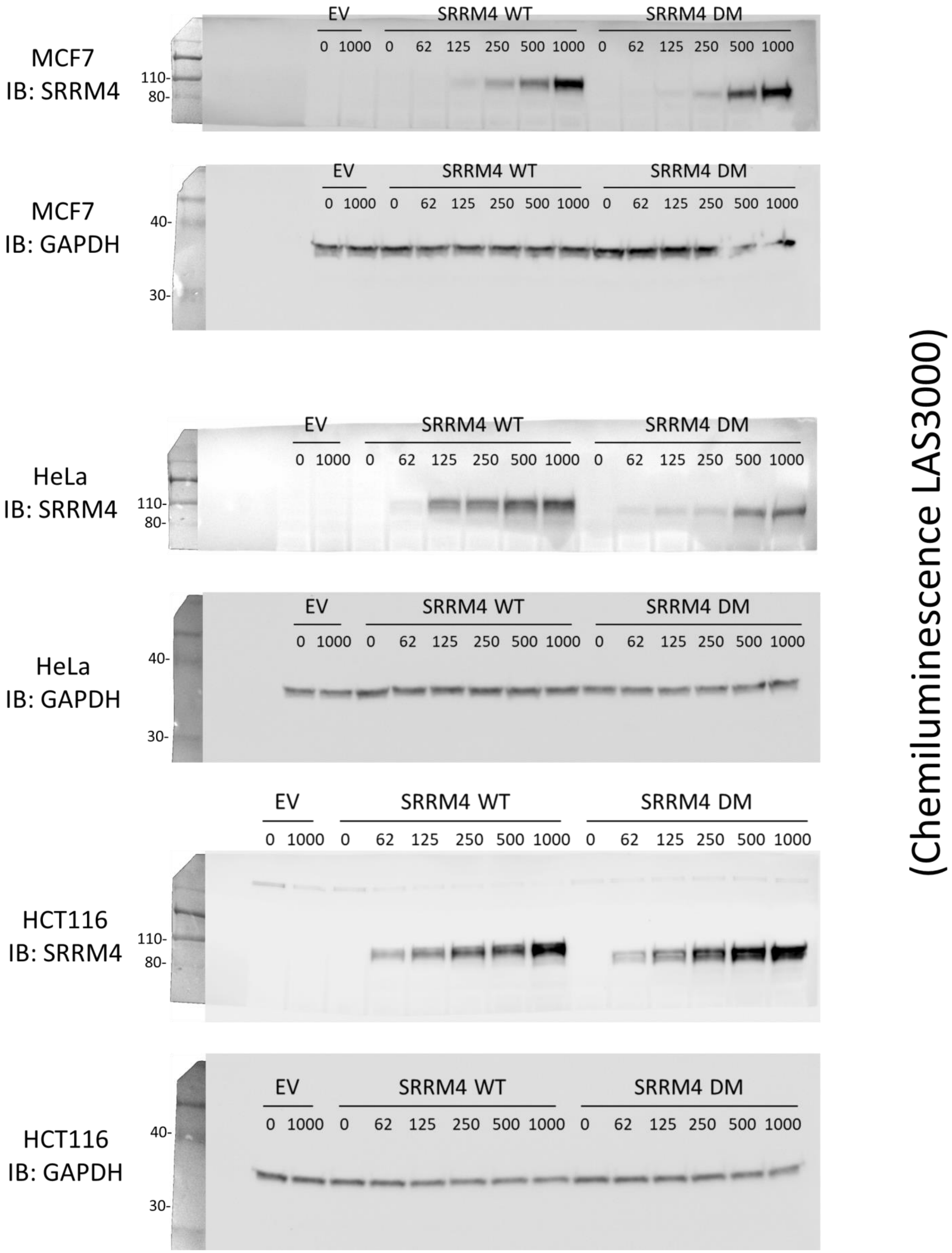
MCF7
IB: SRRM4
EV
SRRM4 WT
SRRM4 DM
0 1000 0 62 125 250 500 1000 0 62 125 250 500 1000
110-
80-
MCF7
IB: GAPDH
40-
30-
HeLa
IB: SRRM4
HeLa
IB: GAPDH
HCT116
IB: SRRM4
HCT116
IB: GAPDH
(Chemiluminescence LAS3000)

S7 Fig

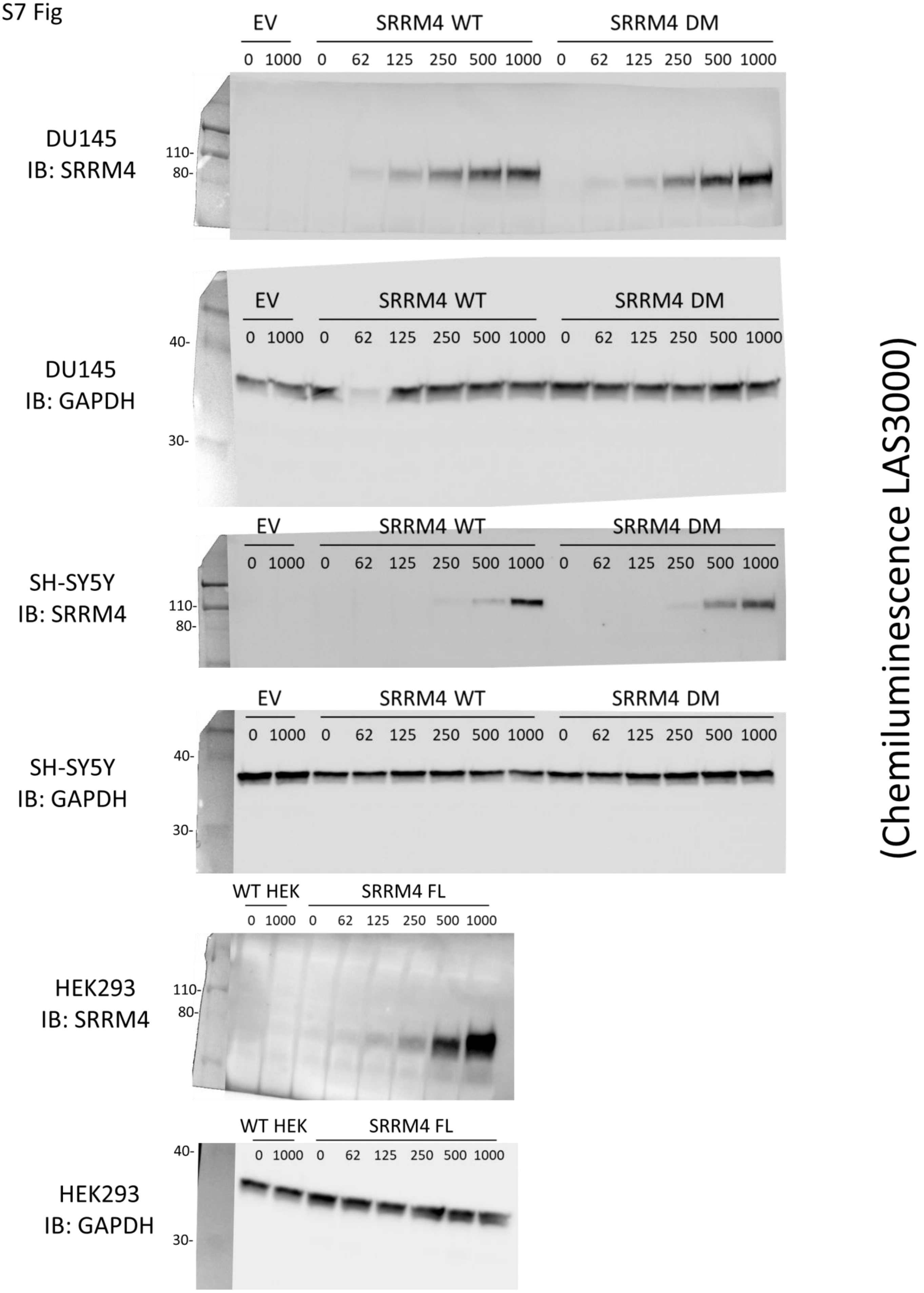

EV
SRRM4 WT
SRRM4 DM
0 1000 0 62 125 250 500 1000 0 62 125 250 500 1000
DU145
IB: SRRM4
110-
80-
EV
SRRM4 WT
SRRM4 DM
0 1000 0 62 125 250 500 1000 0 62 125 250 500 1000
DU145
IB: GAPDH
40-
30-
EV
SRRM4 WT
SRRM4 DM
0 1000 0 62 125 250 500 1000 0 62 125 250 500 1000
SH-SY5Y
IB: SRRM4
110-
80-
EV
SRRM4 WT
SRRM4 DM
0 1000 0 62 125 250 500 1000 0 62 125 250 500 1000
SH-SY5Y
IB: GAPDH
40-
30-
WT HEK
SRRM4 FL
0 1000 0 62 125 250 500 1000
HEK293
IB: SRRM4
110-
80-
WT HEK
SRRM4 FL
0 1000 0 62 125 250 500 1000
HEK293
IB: GAPDH
40-
30-
(Chemiluminescence LAS3000)
