# Supplementary material for: Silencing of SRRM4 suppresses microexon inclusion and promotes tumor growth across cancers
Source: PLoS Biol. 2021 Feb 23;19(2):e3001138. doi: 10.1371/journal.pbio.3001138 (PMC7935315; doi:10.1371/journal.pbio.3001138)
Supplement: Attachment — Submitted filename: Response to reviewers.docx [file pbio.3001138.s036.docx]

Reviewer #1:

This is an interesting manuscript which identifies that the splicing factor SRRM4 is greatly silenced in tumor cells compared to normal cells. SRRM4 is a regulator of microexon inclusion and inclusion of SRRM4 target exons is generally downregulated in tumors. These results are novel.

We are grateful to the reviewer for recognizing the novelty of our work. We hope that we have sufficiently addressed each of the concerns in our point-by-point responses below and the corresponding changes to the resubmitted manuscript.

The major weakness of the manuscript are as follows:

1)	It is not clear why altering SRRM4 levels and microexon inclusion would alter tumor cell growth. Are there specific microexons whose inclusion are critical to the growth regulatory effects of altering SRRM4 levels?

We agree with the reviewer that the mechanistic link between altering microexon inclusion levels and affecting tumor cell growth should be expanded upon. As we discuss in the manuscript, the fact that we detect no single microexon changing significantly in all of the 9 tumor types suggests that the antiproliferative activity of SRRM4 is likely mediated by a combined effect of multiple changing microexons. We have now gone further to investigate the functions of the genes with differentially spliced microexons, and added a section to the manuscript highlighting several specific examples in genes with known roles in tumor cell growth and cancer progression, as follows:

*“Among the microexons identified with differential splicing in both the tumors and cell lines, several have reported functions in proliferation and tumorigenesis. For example, TEAD2 is a transcription factor that regulates gene expression downstream of the hippo signaling pathway, a key regulator of cell proliferation that is frequently dysregulated in cancer [49]. The inclusion of a 12 nt exon in TEAD2 (event ID: HsaEX0064372) is one of the most consistently decreased microexons in tumors (6/9 tissue types in our dataset; S12a Fig) and is SRRM4 regulated, as evidenced by its upregulation in all 6 cancer cell lines after SRRM4 overexpression (S12b-c Fig). Although the function of this particular microexon has not been studied, inclusion of a paralogous microexon in TEAD1 has been shown to disrupt DNA binding and thereby inhibit cell proliferation in hepatocytes [50]. Other examples of cancer-related genes with differentially spliced microexons include SPTAN1 (S13 Fig), a ubiquitously expressed cytoskeletal protein which has recently been shown to affect tumor growth by enhancing cell proliferation and migration [51]; DOCK7 (S14 Fig), which regulates the RAGE-Cdc42 pathway that promotes cancer progression [52] and glioblastoma cell invasion [53]; and PSAP (S15 Fig), which has been implicated in the tumorigenesis of glioblastoma [54], prostate [55,56] and breast cancer [57]. In support of possible antiproliferative roles for these exons, we find significant negative correlations between their inclusion and mitotic index across multiple tumor types (S2 Table). Thus, microexon splicing changes affecting the activity of multiple genes with known roles in cancer likely contribute to the antiproliferative phenotype of SRRM4. Our ongoing work aims to identify the roles of individual microexons in proliferation and tumorigenesis.”*

2)	It would be helpful to show a few examples of splicing events (eg by RNA-seq coverage plots, Sashimi plots, or RT-PCR for example) regulated by SRRM4 in the cell line experiments performed here.

As suggested by the reviewer, we now show the differential inclusion of several top SRRM4-target exons in the cancer cell lines by RT-PCR. We chose 6 exons that are both induced by WT SRRM4 in all 6 cell lines and also show decreased inclusion in multiple tumor types. The PCR results are included in the revised manuscript as **S12-S17 Figs** in the revised manuscript (panel c), alongside plots of the microexon PSIs in the TCGA datasets (panel a) and the RNA-seq data from the cell lines (panel b).

3)	Can the authors demonstrate that methylation of the SRRM4 promoter reduces SRRM4 mRNA/protein levels?

To address this question, we performed a correlation analysis of SRRM4 expression with promoter methylation across tumor samples from TCGA. We find a significant negative correlation between expression and methylation (R_Spearman_ = -0.36), as shown below in **Fig 1 of this letter**. This negative correlation also holds up for every tissue type analyzed separately (**Table 1 of this letter**, below). Therefore, we conclude that increased methylation of SRRM4 promoters can be considered to reduce SRRM4 mRNA levels.

We also attempted to compare SRRM4 methylation/expression with SRRM4 protein levels using the proteomics datasets available from TCGA but, unfortunately, SRRM4 was not detected in these datasets. However, the association between mRNA and protein levels is more generally accepted. In addition, the positive correlations between SRRM4 expression and the PSIs of its target exons (shown in **S4 Fig**) support the assumption that SRRM4 mRNA levels do change the amount of functional protein expressed.

**Fig 1: Correlation between SRRM4 promoter methylation and expression across tumor samples.**

| Tissue: | breast | colorectal | headandneck | kidney | liver | lung | prostate | thyroid | uterus |
| --- | --- | --- | --- | --- | --- | --- | --- | --- | --- |
| R_Spearman_ | -0.132 | -0.408 | -0.173 | -0.131 | -0.046 | -0.285 | -0.418 | -0.160 | -0.345 |

**Table 1: Correlation between SRRM4 promoter methylation and expression across tumor samples, separated by tissue type.**

4)	Does downregulation of SRRM4 in non-malignant, non-neuronal cells alter their growth?

We agree with the reviewer that the ideal experiment to support our model would be to demonstrate that downregulation of SRRM4 increases cell growth. We did attempt the suggested experiment in different cell lines, but we obtained contradictory results using three different shRNA sequences (one increased proliferation rate, one decreased proliferation rate, and one killed all the cells). We believe this is likely because the expression is very low and therefore the shRNA could be hitting off targets.

Instead, to demonstrate that the small differences we detect in SRRM4 expression can have an impact on cell growth, we designed a new experiment to mimic the growth competition between cells with high and low levels of SRRM4, as would occur *in vivo* during tumor evolution. In this experiment, MDA-MB-231 cells with inducible expression of either WT or DM SRRM4 were mixed at a 1:1 ratio. The co-cultures were then passaged for several weeks in the presence of serial dilutions of doxycycline, such that the lowest concentration (31.3 ng/mL) achieved a similar level of SRRM4 induction as the change in SRRM4 expression observed between tumors and healthy tissues (2.6-fold, **S10a Fig** in the revised manuscript). Samples of the co-cultures were taken weekly, and the proportion of each cell line in the population was monitored by PCR using primers flanking the deleted region of SRRM4, allowing cells expressing WT or DM SRRM4 to be differentiated by the size of the PCR product. Compared to the ratio of WT to DM cells at day 1, we observed a dose- and time-dependent decrease in the WT/DM ratio that was significant after 2 weeks of co-culture at all concentrations tested (**Fig 5e-f** in the revised manuscript). We also performed the same experiment in HCT116 cells and observed similar but even stronger effects (**S11 Fig** in the revised manuscript), although the levels of SRRM4 induction were higher in this cell line with the same concentrations of doxycycline (**S10b Fig** in the revised manuscript).

These results demonstrate that the changes in SRRM4 expression observed between tumors and normal tissues are sufficient to provide a growth advantage to tumor cells with lower functional SRRM4 expression. We would contend that the assay described above demonstrates the same concept as downregulation of SRRM4, as we use a cancer cell line with very low basal SRRM4 expression to express physiologic levels of either WT SRRM4 (analogous to normal tissue) or an inactive mutant of SRRM4 (analogous to tumor tissue).

5)	The suggestion that altering SRRM4 levels in somatic cancer cells makes them "neuron-like" seems to be a stretch for non-neuronal cells. The idea that a transformed epithelial will grow projections that having anything to do with axons by altering SRRM4 levels is hard to believe. This point needs to be softened in the manuscript.

We understand the concern of the reviewer and agree that the morphological changes we observe in SH-SY5Y neuroblastoma cells, while qualitatively similar to those seen during published *in vitro* differentiation protocols with this cell line, are not necessarily indicative of true axons or dendrites. Our intention was to highlight that the morphological changes in SH-SY5Y cells after SRRM4 overexpression are consistent with the shift in gene expression and splicing patterns towards that of differentiated neurons (as shown in **Fig 7e**), as well as the functional enrichment of the differentially spliced SRRM4 targets in GO terms such as “plasma membrane bounded cell projection organization” and “cell morphogenesis involved in neuron differentiation” (as shown in **Fig 4a**). Nevertheless, we agree that the point should be softened in the manuscript, and have therefore replaced references to “dendrites” with “projections” and modified other sections of text as follows:

Results section title: *“SRRM4 expression induces neuronal-like expression, splicing patterns, and morphology in cancer cells”* to *“SRRM4 expression induces neuron-like expression and splicing patterns in cancer cells”*

Fig 7 legend: *“WT SRRM4-expressing cells develop numerous projections ~~indicative of neuronal differentiation~~ (orange arrows).”*

*“We also observed striking morphological changes in SH-SY5Y upon SRRM4 overexpression, where cells developed numerous ~~dendrite-like~~ membrane projections after 1 week of induction”*

*“...expression of SRRM4 in cancer cells leads to an upregulation of microexon inclusion, neuron-like expression and splicing patterns, and neuronal morphology...”* to *“...expression of SRRM4 in cancer cells leads to an upregulation of microexon inclusion and neuron-like expression and splicing patterns...”*

---

Reviewer #2:

Head et al. investigate the role of SRRM4, a neuronal splicing factor that regulates micro-exons, in human cancers. Although SRRM4 has been implicated in neuronal tissue development and in neuronal diseases, whether SRRM4 can contribute to disease pathogenesis in non-neuronal tissue was unknown. Here, the authors analyze RNA-seq data from >6,900 TCGA cancer patients and demonstrate that SRRM4 is expressed at lower levels and is hypermethylated in tumor vs. adjacent normal tissues. They identify a small set of SRRM4-regulated micro-exons that are differentially spliced in several tumor types. They show that overexpression of SRRM4 WT but not of a deletion mutant decreases growth of cancer cell lines in vitro, as well as tumor xenograft growth in vivo in a breast cancer murine model. Finally, they uncover a set of exons more included across multiple cancer cell lines with SRRM4 overexpression.

Overall, the findings presented here are interesting, represent a considerable amount of work, and provide the first evidence of the molecular role of SRRM4 mutants in non-neuronal tissues. However, the experimental design, computational and statistical analysis are insufficiently described, making it difficult to fully grasp the validity of some of the study findings.

We thank the reviewer for recognizing the novelty and effort behind our work. We apologize for the issues raised regarding insufficient descriptions of experimental design and computational and statistical analyses. We have done a thorough re-reading of the text to identify and correct these mistakes, and we hope that they have been appropriately addressed in the revised version of the manuscript.

In addition, several key questions should be addressed before the proposed research is ready for publication. Importantly, the paper is based on the observation that SRRM4 transcripts are expressed at lower levels in tumors vs. normal tissue, that relies on data that most scientists would consider as noise in RNA-seq experiments. Therefore it is critical that the authors demonstrate that this level of change in transcript expression leads to protein changes in tumors and has a functional consequence in cell lines. Their experiments use overexpression at >20-100 fold protein changes that are likely not to be physiologically relevant given the baseline SRRM4 expression in tumors and normal tissues.

We understand the concerns of the reviewer that the SRRM4 expression levels are in a low range, and that changes observed in this range might be considered unlikely to have a significant physiological consequence. We believe that our point-by-point responses to the comments below, along with the new experiments performed and additions to the manuscript text, will provide sufficient evidence to prove that our findings of decreased SRRM4 expression in cancer are both quantitatively reliable and physiologically relevant.

Major comments:

1)	As stated by the authors and shown in Fig. 2, SRRM4 transcripts are expressed at very low levels in non-neuronal tissues (median RSEM expression ~4 in normal tissues, ~0.6 in tumors). Those values are typically filtered out from a differential expression analysis because of the low confidence of these reads which are usually considered as noise. As a comparison median expression of SSRM1 transcripts is ~1,500, SSRM2 ~10,000, SRRM3 ~50 RSEM counts This raises the question of the sensitivity and accuracy of SRRM4 transcripts detection using RNA-seq data, especially considering that TCGA data has overall rather poor read coverage. Therefore, one wonders if these small changes in SRRM4 transcript levels can actually have any physiological consequences and lead to detectable changes in SRRM4 protein expression in tumors vs. normal tissues. This is a critical question as all the paper relies on this assumption.

Please note that we have divided the responses to each of the reviewer’s major comments into numbered sections where applicable (e.g. **1a, 1b, 1c**) for ease of reference.

**1a)**	We agree with the reviewer that lowly expressed transcripts need to be treated with caution when performing differential expression analysis. However, we would like to clarify several points in support of the accuracy of our analysis:

1. It has been shown previously that RNAseq is sensitive enough to accurately quantify gene expression levels at values similar to those of SRRM4 in these samples (e.g. PMID: 24454679).
2. The datasets analyzed in this study contain large numbers of samples (n is in the range between 35 and 1135 with a mean of 384 samples per group; see **S1 Table**) resulting in a very high statistical power to detect differential expression between the tumor and normal groups.
3. We detect differential expression using a non-parametric Wilcoxon-Mann-Whitney test, which has been shown to have very low false positive rates on simulated expression data even under high noise level conditions (PMID: 12424116).
4. The coverage of TCGA samples ranges from 53 million to 140 million reads (with a mean of 79 million; only considering read pairs mapping to human genes). This is in fact in line with illumina recommendations for RNAseq experiments ("Experiments looking for a more global view of gene expression, and some information on alternative splicing, typically require 30–60 million reads per sample"; source: https://support.illumina.com/bulletins/2017/04/considerations-for-rna-seq-read-length-and-coverage-.html) and are well beyond saturation levels of statistical power to detect differential expression given the high number of samples in each group (PMID: 24319002).
5. We see a consistent downregulation of SRRM4 across all tumor types tested (excluding liver, which has zero expression in normal tissue) (**Fig 2a**). We also note that if the change in SRRM4 expression was due to noise, there would be no implicit reason it should consistently change in the same direction across different tissue types, or within different samples in the same tissue type. This is supported by the fact that other lowly expressed splicing factors do not show the same consistency in the direction of change (**Fig 2** **of this letter**, in response to comment 1e).

Taken together, we believe these points provide substantial evidence that we can confidently detect SRRM4 differential expression, and that these differences are not due to noise. In fact, consistently regulated “noise” is not noise but signal by definition, and our observation of strikingly consistent downregulation of SRRM4 in tumors, even at low levels, supports our hypothesis. While we cannot detect SRRM4 protein directly from TCGA datasets without doing targeted proteomics experiments, the positive correlations between SRRM4 expression and the PSIs of its target exons (shown in **S4 Fig**) support the assumption that SRRM4 mRNA levels do change the amount of functional protein expressed and have an impact on the splicing patterns observed in cancer.

The authors compare expression in tumors vs. normal tissues, yet the details of how the differential analysis was performed or what is the magnitude of fold change are not provided; these details should be provided.

**1b)**	A detailed description of how the differential analysis was performed is included in the Methods section under the heading “Statistical Information”:

*“Differential expression/methylation of splicing factors and differential exon inclusion were determined by two-tailed unpaired Wilcoxon-Mann-Whitney test, using the Benjamini Hochberg method to correct for multiple testing. The numbers of samples per group are indicated in S1 Table, and p- and q- values are reported in S1-3 Data.”*

For clarity, we have now included a reference to this information within the main text as follows:

*“Remarkably, the median expression levels of SRRM4 were significantly lower in tumors from all tissues, with the exception of liver that had median expression of 0.0 in both tumor and normal samples (p < 0.001; Wilcoxon-Mann-Whitney test;* ***Fig 2a, S1 Data****. Details of the statistical tests are found in the Methods section under “Statistical Information”).”*

The magnitude of fold change for SRRM4 expression is represented in **Fig 2e**, and the exact median expression values in tumor and normal tissues can be found in **S1 Data**. For convenience, we have also modified **S1 Data** to include a new column “fold change (tumor/normal)” where this value is calculated directly.

We also note that the change in methylation levels, which in the previous submission was also calculated as fold change, should be more accurately calculated as a difference because it represents a fraction of methylation (β-value) between 0 and 1. Therefore, **Fig 2g** has been updated accordingly, as well as **S2 Data**, where the difference between tumor and normal is now included as a new column.

Fig 2A should also indicate whether the data is plotted as median or mean, and what the quartile ranges are.

**1c)**	We have now added the following description to the legend of **Fig 2a**:

*“Boxes expand from the first to the third quartile, with the center values indicating the median. The whiskers define a confidence interval of median ± 1.58*IQR/√(n).”*

Additionally, the authors claim that other SRRM genes (SRRM1-3) are not differentially expressed in tumors. Yet the data presented in Dataset 1 show significant P-values, even lower than for SRRM4, for SRRM1-3 transcripts in many other tumors types. The authors need to clarify this discrepancy between the data presented in the Fig 2C and Dataset 1.

**1d)**	We apologize for the lack of clarity on this point. The reviewer is correct that other SRRM genes do show significant differential expression in several tumor types; however, unlike SRRM4, these changes are not consistent across all tumor types. For example, SRRM3 expression changes significantly in 6/9 tumor types, decreasing in 2 and increasing in 4. As shown in **Fig 2c** of the manuscript, where each point on the plot represents the tumor/normal ratio of SRRM gene expression for one tissue type, only for SRRM4 does this ratio change significantly and in the same direction for all tissues.

To clarify this point, we have rewritten the sentence in the manuscript text:

*“Other members of the SRRM family (SRRM1-3) did not show the same pattern of consistent change (significant and in the same direction) across tissues at either the expression or the promoter methylation level (Fig 2c, S1-2 Data).”*

Given the low baseline SRRM4 transcripts expression, even a slight change in counts would be accounted as a major expression change. Therefore, for Fig. 2D, one wonders if SRRM4 is the top hit because if its low expression. We recommend the authors demonstrate that other splicing-factor genes expressed at low levels (within the same RSEM values) in non-neuronal tissues do not exhibit expression differences between tumors vs. normal tissues.

**1e)**	As suggested by the reviewer, we have analyzed the changes in expression of lowly expressed splicing factor genes (all SFs with median RSEM < 5 for either tumor or normal group, excluding 0’s) compared to all splicing factor genes or SRRM4 alone. The results of this analysis are shown below in **Fig 2 of this letter**. We found that the average tumor/normal ratio for lowly expressed genes was 1.1, which was comparable to that of all splicing factor genes (1.133) and not significantly different from 1 by one-sample t-test (p = 0.5911). In contrast, SRRM4 had an average ratio of 0.3142 across tissues, which is significantly different from 1 by one-sample t-test (p < 0.0001). Therefore, we conclude that there is no general bias towards decreased expression in tumors among lowly expressed splicing factors.

**Fig 2: Lowly expressed splicing factors do not show a bias in the direction of change between tumor and normal tissue samples**. Error bars represent standard deviation.

2)	Although SRRM4 is mostly expressed in neuronal cell types, the author focus their analysis on non-neuronal tumor types. Yet they state "the most striking decrease was observed in glioblastoma, likely due to the higher inclusion of SRRM4 targets in glia than other normal cell types", suggesting that SRRM4 might play a role in tumors of neuronal origin. Given the availability of RNA-seq data from neuronal tumors in TCGA, as well as normal samples in GTEX, the authors should explain why they did not investigate the role of SRRM4 in these tumors. The author state they use RNA-seq data from TCGA for which paired normal samples are available and therefore would exclude neuronal TCGA tumors. Yet since they do not performed paired analysis for each patient, they could very well include data from normal GTEX neuronal tissues, which would permit the analysis of neuronal tumor types for which no TCGA data from normal tissue would be available.

As the reviewer points out, we did not include an analysis of glioblastoma data from TCGA because of the lack of sufficient normal tissue samples for comparison (only 5 normal brain samples are available from TCGA, and we only selected tissues with at least 20 samples per group). However, the suggestion to compare glioblastoma samples from TCGA with normal brain samples from GTEx is feasible. In fact, a similar analysis has been published since the initial submission of our manuscript, using a different computational method to quantify differential exon inclusion from a number of normal brain, glia and glioma datasets, including those from TCGA (glioblastoma) and GTEx (normal cortex and hippocampus) (Reixachs-Sole et al. 2020, PMID: 32286305). Across these datasets, the authors consistently found a decrease in microexon inclusion in glioma compared to the glia and normal brain samples. These results are in close agreement with our findings using glioblastoma cell lines from the NCI-60 and normal glia samples from VastDB (now removed from the manuscript, due to the reviewer’s criticism in minor point 5) and are consistent with the overall model of microexon downregulation in cancer in general. That said, we would like to point out that a difference in microexon inclusion should be expected between glioblastoma and normal brain tissue samples, due to the latter containing a higher proportion of neurons. For this reason, we would argue that a more appropriate comparison is between glioblastoma and normal glia samples, as shown by Reixachs-Sole et al.

We have now included a sentence highlighting the findings from Reixachs-Sole et al. 2020. in the revised manuscript:

*“Taken together, these results suggest that the observed downregulation of microexon inclusion is a general phenomenon across a wide variety of cancer types. Indeed, a recent study of splicing changes between glia and glioblastoma also reported decreased microexon inclusion in glioblastoma [23], which is consistent with our findings in tumors from non-neural tissues and further suggests the generalizability of these findings to different cancer types.”*

3)	The authors focus on a set of spliced isoforms regulated by SRRM4 in neuronal cell types (Fig. 3). Whether these exons are relevant in non-neuronal cells remains to be determined. First, the authors should explain where does the list of SRRM4 targets comes from?

**3a)**	Our list of SRRM4 targets was derived from an overexpression experiment in HEK 293 cells, using a stringent cutoff of ΔPSI ≥ 25 to include only high confidence targets (314 exons in total). This experiment was performed before initiating any of the bioinformatic analyses and was therefore our “gold standard” list at the beginning of the study. However, one of the two replicate RNAseq experiments used to generate our list was not previously published, and therefore the list and the raw data are included in the supporting information of the current study (**S4 Data**, SRA accession SRX4193731 and SRX4193721).

A description of the above was included in the methods under the heading “*Correlation between SRRM4 activity and target exon PSI levels*”, but for clarity we have now created a separate methods section called “*Definition of SRRM4 target exon list*” where this description can be found more easily.

The authors claim that tumor spliced exons are "significantly enriched" in SRRM4 targets. How many of these are micro-exons?

**3b)**	As shown in **S3 Fig**, a majority (55.5%) of differentially included SRRM4 targets are ≤ 27 nt, and these represent a greater proportion of the SRRM4 targets decreasing in tumors (63%) than of those increasing (39%). To more clearly represent these results visually, we have modified the histogram of differentially spliced SRRM4 exons shown in **Fig 3a** of the manuscript (red/blue bars), with exons ≤ 27 nt shown in brighter colors and those > 27 in light colors. We further repeated the statistical enrichment analysis in differentially spliced exons considering only SRRM4 targets ≤ 27 nt (the length of a microexon as defined in our study) and found that the enrichment remains significant for 5/9 tissue types when compared against a background of all significantly changing exons.

Fig. 3A seems to indicate <50 SRRM4 targets are spliced-out in tumor vs. normal, which is not very impressive, especially given that none of them is detected across all tumor types.

**3c)**	Our analysis identified 84 SRRM4 targets (out of 314) that are differentially spliced in at least 1 tumor type. Of these, 71 show decreased inclusion in at least 1 tumor type, while 37 show increased inclusion. However, as shown in **Fig 3b**, around 2/3rds of SRRM4 targets are neural-specific, meaning they have 0 inclusion in normal non-neural tissues, and therefore they cannot possibly decrease further in tumors. For this reason, we would argue that it is impressive that so many more SRRM4 targets are found to decrease in tumors than to increase, as almost every SRRM4 target that can decrease (i.e. has basal inclusion > 5% in non-neural tissues) does decrease (red points in **Fig 3b**).

Finally, the reviewer is correct that none of the SRRM4 exons show differential splicing in every tumor type; however, it should be noted that most exons are not detectable in every tumor type due to variability in expression of the host gene across tissues. In fact, there are exons that change in every tumor type where the exon is detected (e.g. PAPSS2 [HsaEX0045471], with ΔPSI(T-N) > 5 in 6/6 tissue types detected). As described in the methods section, all exons whose quality score in VAST-TOOLS is calculated as VLOW (meaning ≤ 10 reads mapping to the specific splice junctions) are filtered out of our inclusion tables, and those exons with less than 20 samples with sufficient read coverage in both tumor and normal samples in a given tissue type are removed. Therefore, the grey boxes in **Fig 3c** often represent an exon that cannot be quantified, rather than an exon that does not change. To clarify this point, we have changed **Fig 3c** so that exons without quantification in a given tissue type are now shown in grey, and updated the figure legend accordingly.

The authors perform differential splicing analysis on tumor vs. normal samples using a cut-off of >5% splicing change is within the limit of biological and technical noise. Yet, when analyzing data from cell lines they use a cut-off of >25%. We recommend using the same cut-off measuring at least >10% of splicing change differences.

**3d)**	We agree with the reviewer that the choice of a ∆PSI cutoff to define differential splicing events requires a careful balance to maintain signal while minimizing noise. For the TCGA analysis, we chose a ∆PSI cutoff of 5% to include as many relevant events as possible while excluding those unlikely to have any biological significance. It is important to note that these analyses include dozens to hundreds of samples per group, and therefore we are able to minimize noise by applying a statistical cutoff (q < 0.01). We also note that our use of a 5% cutoff is substantially more conservative than other recent studies of global alternative splicing changes in cancer using TCGA data, which use only statistical cutoffs to define alternatively spliced events regardless of ∆PSI (e.g. PMID: 29045845, 30078747).

Nevertheless, to address the concern of the reviewer, we have reanalyzed the TCGA data using a 10% cutoff as suggested. While the total number of differential splicing events detected drops to about 1/3rd of those found with a 5% cutoff (**Fig 3 of this letter**, below), the proportion of SRRM4 targets decreasing in tumors is nearly identical (**Fig 3 and 4 of this letter**, below). In fact, this proportion increases with increasing ∆PSI cutoffs between 10-27%, after which point all changing exons are decreasing in tumors (**Fig 3 of this letter**, below). We therefore conclude that the overall finding of SRRM4 target exons decreasing in cancer is robust to the use of different ∆PSI cutoffs. Because our goal in this study was to uncover global splicing patterns rather than to identify individual relevant targets, we believe that using a 5% cutoff allows us to be more inclusive and identify all potentially relevant targets while keeping the global signal.

**Fig 3: TCGA data analysis using different ∆PSI cutoffs.** The percentage of SRRM4 targets decreasing in cancer (red; left y-axis) increases with increasing ∆PSI cutoff, while the total number of differentially spliced exons (grey; right y-axis) decreases.

**Fig 4. Reproduction of Fig 3a using a 10% ΔPSI cutoff.** Distribution of ΔPSIs of differentially spliced SRRM4-target exons between normal and tumor samples in the TCGA datasets. Only exons with significant changes (q < 0.01, |ΔPSI| ≥ 10) are shown. Microexons are highlighted in brighter red (down in tumor) and blue (up in tumor).

For the cell line data, we used a cutoff of 25% to be consistent with the previous analysis in HEK 293 used to generate the SRRM4 target list (as explained above in response to major point 3) and other published studies using cell line data (e.g. PMID: 23739326). We agree with the reviewer that ideally the cutoffs should be consistent between the two sets of analyses. However, unlike the TCGA data, the cell lines only have 1-2 replicates per sample, so it is not possible to use a statistical cutoff to reduce biological and technical noise. Therefore, the use of a higher ∆PSI cutoff ensures that the detected exons are only high confidence SRRM4 targets. Furthermore, it is to be expected that the splicing changes in vitro with SRRM4 overexpression will be larger than those seen in patient samples, where hundreds of molecular changes are occurring that may mask the effects of one splicing factor.

We have also reanalyzed the cell line data using different ∆PSI cutoffs. Predictably, the number of exons increasing in all 6 cancer cell lines with expression of WT SRRM4 increases substantially with decreasing ∆PSI. Notably, the overlap with exons decreasing in tumors remains statistically significant at 5%, 10%, or 25% for all 9 tissue types (**Table 2 of this letter**, below). We therefore conclude that the ∆PSI cutoffs used do not impact the overall findings of the study, neither in defining the exons that change in tumors nor their overlap with those changing in the cancer cell lines.

| **Tissue** | ∆**PSI 5** | ∆**PSI 10** | ∆**PSI 25** |
| --- | --- | --- | --- |
| **Total # exons detected** | **363** | **245** | **131** |
| breast | 3.14E-20 | 1.07E-29 | 4.21E-14 |
| prostate | 3.98E-09 | 6.55E-21 | 7.23E-10 |
| kidney | 3.79E-19 | 2.33E-19 | 0.000203 |
| lung | 3.97E-17 | 8.26E-23 | 3.69E-16 |
| headandneck | 3.68E-12 | 4.65E-14 | 4.66E-09 |
| uterus | 1.06E-12 | 7.76E-18 | 1.06E-06 |
| liver | 0.000312 | 5.98E-08 | 0.000721 |
| thyroid | 4.28E-11 | 4.80E-08 | 2.21E-05 |
| colorectal | 3.58E-19 | 1.33E-30 | 1.81E-13 |

**Table 2:** Significance of the overlap between exons decreasing in tumors from TCGA data (∆PSI > 5) and exons increasing in all 6 cancer cell lines after overexpression of WT SRRM4 using different ∆PSI cutoffs (5, 10, and 25). P-values shown are calculated using a one-sided fisher exact test.

Given the known genomic intra- and inter-tumor heterogeneity, and since Fig. 2A indicates widespread differences in SRRM4 transcripts levels within tumors, the authors could have divided the tumors into low- and high-SRRM4 expression, and compared the splicing targets within these two classes. This approach could potentially additional SRRM4 targets that are not diluted in a mixture of low- and high-SRRM4 expressing samples.

**3e)**	We thank the reviewer for the idea, and as suggested have performed an analysis where we stratify patients based on SRRM4 expression. For each cancer type, we split tumor samples into those with SRRM4 expression smaller or equal to the median and those where expression was higher than the median. Applying the same test and criteria as was done previously for differential exon inclusion between healthy and tumor samples, we identified differentially included exons between SRRM4-high and -low tumor samples in 5 out of 9 cancer types (|ΔPSI| > 5 and q < 0.01), although the total number of exons identified was lower (as expected because SRRM4 expression variation between healthy and tumor samples is higher than within tumor samples). In all cases, a majority of the identified exons were also identified as being differentially spliced between normal and tumor samples. Of those exons not identified previously, none showed differential splicing between SRRM4-high and -low groups in more than one tissue, suggesting that these newly identified exons might not be high-confidence SRRM4 target exons. Therefore, we kept the focus in our manuscript on the analyses of differentially included exons between normal and tumor samples.

Also, the authors should consider the possibility that these spliced isoforms can be regulated by other splicing factors, for example SRRM3 which regulate very similar targets to SRRM4 (Gonatopoulos-Pournatzis et al. 2018), as well as SRSF11, RBFOX or PTBP1 which also regulate micro-exons (Gonatopoulos-Pournatzis et al. 2018, Li et al, 2015). Whether these genes are differentially expressed along with SRRM4 in tumors vs. normal tissues should be determined. Finally, given the fact that 2-fold changes in SF-levels are sufficient to lead to splicing changes, the authors should at least discuss the possibility that other SFs, including SRNPB, RBM34, RNPS1, ELAVL1 an RALYL, could contribute to tumor-associated splicing events in the analyzed datasets.

**3f)**	We agree with the point of the reviewer that we cannot rule out that changes in other splicing factors contribute to the tumor-associated splicing events observed in the analyzed datasets. To address this concern, we have included the following discussion of this topic in the revised manuscript:

*“Other splicing factors aside from SRRM4 have been shown to be involved in the regulation of microexon splicing, including SRRM3, SRSF11, RBFOX, and PTBP1 [19,22,46]. While SRRM4 is the only one of these factors that changes significantly and in the same direction across all tumor types included in our study, others also display significant expression changes in specific tumor types (S1 Data). Therefore, we cannot rule out their potential involvement in the regulation of individual microexons in certain tissues. Interestingly, our results suggest that SRRM3 is not involved, despite being thought to regulate similar targets as SRRM4, as its expression is increased in a majority of tumor types (Fig 2c). This supports the idea of differing roles for SRRM3 and SRRM4 in vivo, as has been previously proposed by others [47]. In addition to the factors mentioned, it is likely that other SFs identified here as having consistent changes across all tumor types (SNRPB, RNPS1, RBM34, ELAVL1, RALYL; Fig 2e,g) could contribute to tumor-associated splicing events in the analyzed datasets. Due to the high degree of splicing dysregulation in cancer generally, as well as the complex crosstalk between splicing factors which often regulate each other’s activity [14,48], it is very difficult to attribute observed splicing changes in cancer to one specific factor. Nevertheless, our findings of consistently decreased SRRM4 expression across tumor types (Fig 2a), coupled with the correlation of SRRM4 expression with inclusion of its target exons in tumors (S4a Fig), and their significant overlap with exons increasing after SRRM4 overexpression in different cancer cell lines, provide strong evidence that SRRM4 dysregulation contributes substantially to the observed splicing changes in cancer.”*

4)	Does SRRM4 regulate other exons that are not micro-exons? The author imply this but don't say it. Isn't it possible that these non-microexons are contributing to phenotype? The splicing analysis in tumors should highlight separately the findings from micro-exons and non micro-exons in Fig 3. Similarly, one wonders if the gene enrichment analysis in Fig 4 relates to all SRRM4 targets or just microexons?

We apologize for the lack of clarity on this point. The reviewer is correct that SRRM4 regulates exons that are not technically microexons. As shown by Irimia et. al. (PMID: 25525873), SRRM4-regulated exons have a strong tendency to be smaller than other alternative exons, which average ~140 nucleotides. While we define microexons here as exons ≤ 27 nucleotides in length, it is relevant to note that the exact definition of microexons vs. non-microexons depends on an arbitrary cutoff. By our definition, 61.3% of SRRM4 targets are microexons. Others have used a cutoff of 51 nucleotides (e.g. PMID: 25524026), by which definition 86.6% of SRRM4 targets would be considered microexons, while > 99% of SRRM4 targets are smaller than average (the distribution of exon lengths is shown in **Fig 5 of this letter**, below).

**Fig 5: Histogram of SRRM4 target exon lengths.**

To assess the potential contributions of microexons vs longer SRRM4 target exons to the antiproliferative phenotype, we analyzed separately the correlations of these exons PSIs with mitotic index (as shown combined in **Fig 4d** of the manuscript). We find that those with negative correlations are more likely to be microexons, while those with positive correlations are more likely to be non-microexons (**S5 Fig**). This finding, combined with the fact that a majority of SRRM4 targets are microexons in general, suggests that changes in microexon splicing contribute more to the antiproliferative phenotype than non-microexons. However, we cannot rule out that larger SRRM4 target exons contribute to the phenotype observed. We have summarized these results in the text as follows:

*“Furthermore, a majority of the SRRM4 target exons with significant negative MI correlations were ≤ 27 nt in length, while those with positive correlations tended to be longer (S5 Fig). This finding suggests that SRRM4 target microexons may contribute more to the antiproliferative phenotype than longer SRRM4 target exons; however, we cannot rule out the possibility that longer SRRM4 targets also contribute.”*

In addition, as suggested by the reviewer, we now highlight microexons (≤ 27 nucleotides) in both **Fig 3a and c**. In both cases, it is clear that a majority of differentially spliced microexons have decreased inclusion in cancer. We have also included a new figure (**Fig 3d** of the revised manuscript) showing the percentages of differentially spliced microexons that decrease in each tumor type, which comprise a majority in each case and range from 60-87% depending on the tissue. Similarly, the GO enrichment analysis in **Fig 4a** relates to all SRRM4 targets, but repeating the analysis with only SRRM4 target microexons gives similar results (**Fig 6 of this letter**, below).

**Figure 6: GO term enrichment of genes with downregulated microexons in tumors from TCGA** (GO biological process complete).

Thus, we conclude that the overall findings of our study hold when looking exclusively at microexons. However, because the majority of our study revolves around changes in SRRM4 expression (both as an observation in the TCGA data and in our experiments with cancer cell lines), we felt it more appropriate to report the findings on splicing changes of SRRM4 target exons taken as a whole.

5)	In Fig 4, the authors correlate SRRM4 transcript expression with the mitotic index, but fail to indicate whether SRRM4 expression correlates with other tumor features, including tumor grade, presence of know cancer driver alterations (e.g., TP53, PTEN, MYC etc), patient overall survival, as well as tumor purity. This last point is particular relevant given high intra-tumor heterogeneity in solid cancers, raising the question whether decreases SRRM4 expression detected in tumor samples is due to changes in stromal or immune cell composition in tumors vs. normal tissues rather than in cancer epithelial cells.

We thank the reviewer for these suggestions. We have now analyzed the correspondence between SRRM4 expression and all other tumor features suggested by the reviewer (shown in **S6 Fig** in the revised manuscript). We report these results in the revised manuscript as follows:

*“We further tested whether SRRM4 expression correlates with other tumor features apart from proliferation. We did not observe any clear trend of SRRM4 expression changes across different TNM tumor stages (S6a Fig), nor was there a significant correspondence of SRRM4 expression levels with patient survival in any tissue type (Kaplan-Meier method, q < 0.05, FDR corrected). There was also no correlation with tumor purity, as measured by two different methods: ESTIMATE [27] (R_Spearman_ = -0.04) and CPE [28] (R_Spearman_ = 0.038) (S6b Fig). We did detect significant associations between SRRM4 expression and mutations in certain cancer genes, where the presence of mutations in those genes are generally associated with a lower expression of SRRM4. In particular, out of 15 cancer genes tested, 10 of them raised significant associations (p < 0.05), with the presence of 8 of them being associated with a lower SRRM4 expression (S6c Fig). These observations are consistent with the association of most cancer gene mutations with mitotic index, which in 9/10 cases change in the opposite direction as SRRM4 (S6d Fig).”*

6)	In Fig. 5-7, the authors test the consequences of SRRM4 overexpression in cancer cell lines. Do these cell lines all have virtually no SRRM4 protein expression at baseline? It would be nice to see qPCR so that we can compare with the fold changes in tumors vs. normal tissues to the levels of overexpression achieve in the cell lines. If there is no baseline expression at all, then the levels of overexpression are way beyond physiological, and way beyond the changes detected in tumors. Since splicing factors act in dose dependent manner, this raises the question whether this level of expression is physiologically relevant and how it really mimics the changes between tumor and normal tissues. Indeed, in all cell lines, SRRM4 induction with 62 or 125 ng/ml of DOX leads to very mild phenotypic differences. Thus relevance of this data is difficult to grasp in the absence of a quantification of the SRRM4 protein expression in tumors vs. normal tissues.

**6a)**	We understand the concern of the reviewer regarding the physiologic relevance of SRRM4 overexpression levels achieved in vitro. As suggested, we have performed qPCR to assess the levels of SRRM4 overexpression in each cell line after induction with 1 ug/mL doxycycline. The results are shown in **S9 Fig** in the revised manuscript. At this dose, the overexpression ranges from 70- to 500-fold, depending on the parental cell line. Comparing these overexpression levels with the changes seen in tumors, which are on the order of 1.5- to 7-fold, we see that the fold-changes are much higher in the cell lines.

By using varying concentrations of doxycycline in the resazurin growth assays shown in **Fig 5a-c** and **S7 Fig**, we demonstrate the dose-dependence of the effect of SRRM4 expression on cell growth. However, the reviewer is correct that the phenotypic effects at the lowest dox concentrations are mild, making them difficult to quantify during the short time period of these growth assays. In this regard, we hypothesized that the effect of smaller changes in SRRM4 expression on cell growth would be amplified over longer periods of time, as in the case of tumor evolution, which occurs on a timescale of years instead of days. To give a hypothetical example: a 1% increase in the proliferation rate of a tumor cell with a 24-hour doubling time would lead to a 2.8% difference in cell number during the 4 days of our growth assay, making the change difficult to quantify. However, the same difference in proliferation rate would lead to 1288% more cells after a year, thereby completely dominating the population. Therefore, it is logical to surmise that the small changes we observe in SRRM4 expression in tumors have fitness effects that are selected for over long periods of time.

To test this hypothesis experimentally, we designed a new experiment to mimic the growth competition between cells with high and low levels of SRRM4 over time. In this experiment, MDA-MB-231 cells with inducible expression of either WT or DM SRRM4 were mixed at a 1:1 ratio. The co-cultures were then passaged for several weeks in the presence of serial dilutions of doxycycline, such that the lowest concentration (31.3 ng/mL) achieved a similar level of SRRM4 induction as the change in SRRM4 expression observed between tumors and healthy tissues (2.6-fold, **S10a Fig**). Samples of the co-cultures were taken weekly, and the proportion of each cell line in the population was monitored by PCR using primers flanking the deleted region of SRRM4, allowing cells expressing WT or DM SRRM4 to be differentiated by the size of the PCR product. Compared to the ratio of WT to DM cells at day 1, we observed a dose- and time-dependent decrease in the WT/DM ratio that was significant after 2 weeks of co-culture at all concentrations tested (**Fig 5e-f** in the revised manuscript). We also performed the same experiment in HCT116 cells and observed similar but even stronger effects (**S11 Fig** in the revised manuscript), although the levels of SRRM4 induction were higher in this cell line with the same concentrations of doxycycline (**S10b Fig** in the revised manuscript). This result demonstrates that the changes in SRRM4 expression observed between tumors and normal tissues are sufficient to provide a growth advantage to tumor cells with lower SRRM4 expression. Given the relevance of this finding in supporting the overall hypothesis of the study, we have included these results as a main text figure in the revised manuscript.

Furthermore, a western at baseline and one of the DOX concentration for all cell lines on one blot is needed so one can compare the expression levels and the effects on cell growth across multiple cell lines as presented in Fig 5E. The levels look widely different in Fig S5, raising the question whether difference in Fig 5E are due to differences in SRRM4 levels.

**6b)**	The reviewer is correct that there are differences in induction levels between the cell lines, as can be seen in **S9 Fig** in the revised manuscript. Interestingly, these differences do not appear to correlate with the effects on cell growth shown in **Fig 5e** of the original manuscript. For example, HeLa cells, which have the smallest absolute effect in terms of cell growth (maximum inhibition of 30.2% after 4 days of induction with 1 ug/mL doxycycline) had the highest induction of SRRM4 at the RNA level. These discrepancies could be due to a variety of factors that might vary between the different cell lines, including differences in basal proliferation rate, number of viral integrations per cell, efficiency of the doxycycline-inducible promoter, translation efficiency of the induced RNA, RNA/protein degradation rates, etc. As we cannot account for which of these potential differences might influence the magnitude of proliferation inhibition, we agree with the reviewer that the analysis shown in **Fig 5e** of the original manuscript does not have a straightforward interpretation. Since our intention was to show that proliferation is inhibited in all cancer cell lines tested after SRRM4 overexpression, and not to enter into cell-type-specific differences, we have removed **Fig 5e** from the revised manuscript. We believe this change has no effect on the overall conclusions of the manuscript.

Finally, the level of expression of SRRM4 WT and SRRM4 DM are different in Fig 5D, raising the question whether the lack of effect with SRRM4 in vitro and in vivo is due to lower expression.

**6c)**	To address this question, we performed densitometry analysis on the Western blot shown in **Fig 5d** and another experimental replicate. The results are shown in **Fig 7 of this letter**, below. The reviewer is correct that the expression of SRRM4 DM at 1 ug/mL doxycycline is slightly lower than WT at the same concentration (~85% of WT). However, we note that it is still higher than the WT induction level at 500 ng/mL (~60%), at which concentration we still observe significant proliferation inhibition for the WT SRRM4 (as shown in **Fig 5b**). Therefore, we conclude that the lack of effect of the DM SRRM4 on cell proliferation is not due to lower expression.

We also note that in all cell lines, the RNA levels of WT and DM SRRM4 are similar at 1 ug/mL doxycycline, as shown in **S9 Fig**. Therefore, the differing effects of WT and DM SRRM4 on cell proliferation are not due to differences in expression.

**Figure 7: Densitometry of SRRM4 induction levels with different concentrations of doxycycline.** Error bars represent SD from 2 independent experiments.

Finally, the authors should provide RT-PCR or QPCR validations for their top SRRM4-regulated splicing events in cancer cell lines to demonstrate the accuracy of their splicing analysis, and illustrate the false positive detection rate.

**6d)**	As suggested, we have now performed RT-PCR validation of several top SRRM4-regulated splicing events in the cancer cell lines. 6/6 (100%) of tested exons demonstrated increased inclusion in all 6 cell lines as assessed by PCR (**S12-S17 Figs**). We also find a very high correlation (R^2^ = 0.86) between the PSI quantifications obtained by PCR and RNAseq (**Fig 8 of this letter**, below), confirming the overall high accuracy of our splicing analysis.

**Fig 8. Correlation of the PSIs quantified by RNAseq (vast-tools) and PCR (densitometry).**

7)	Comparing expression and splicing changes DOX treated vs. DOX untreated tumor samples can be confounded by DOX-induced expression and splicing as previously described (Ahler et al. 2013, Park et al. 2019). Therefore, events induced by DOX in empty vector control should be removed from the dataset in Fig 7f.

We agree with the concern of the reviewer that splicing changes can occur as a result of doxycycline treatment. However, we did not perform any tumor xenografts with empty vector (EV) control cells, and the RNAseq data generated from the EV cells in vitro were treated with equal concentrations of doxycycline as the WT and DM SRRM4-expressing cells. Therefore, we do not have the data required to perform the exact analysis requested.

As an alternative way to address this concern, we have compared the exons that change between induced and uninduced tumors with those changing between EV and WT-SRRM4 cells in vitro (which are both treated with doxycycline). In this way, we can be sure that the events changing in both datasets are not changing due to doxycycline treatment. As shown in **Fig 9 of this letter** (below), there is a strong positive correlation between the changes in both all microexons (R^2^ = 0.809) and SRRM4-target exons (R^2^ = 0.709) in these two datasets. This result demonstrates that the majority of changes in these exons are not due to doxycycline treatment alone.

**Fig 9: Positive correlation between the ΔPSIs of exons changing with SRRM4 induction in mouse tumors and exons changing between WT-SRRM4 and EV cells in vitro.** Left: SRRM4-target exons (red); right: all microexons ≤ 27 nt (blue).

8)	The author claim that SH-SY5Y cells expressing SRRM4 form "dendrite-like" projections, "reminiscent of SH-SY5Y cells that have undergone neuronal differentiation with retinoic acid (RA) and brain-derived neurotrophic factor (BDNF) treatment" based on a representative bright field picture. Have the authors used any neuronal differentiation cell markers to demonstrate that these are "dendrite-like"?

As suggested by the reviewer, we did attempt to perform immunofluorescence experiments on SH-SY5Y cells using various neuronal differentiation markers; however, none of these produced a detectable signal. Looking at the RNAseq data, we find that although several neuronal marker genes are upregulated in cells with WT-SRRM4 expression compared to the inactive mutant, the expression of these genes remains very low (<1 cRPKM), and thus unlikely to be detectable by IF.

Although it was not our intention to claim that SRRM4 expression causes SH-SY5Y cells to fully differentiate into neurons, we realize that the language used in the manuscript may have given this impression. Our intention was to highlight that the morphological changes in SH-SY5Y cells after SRRM4 overexpression are consistent with the shift in gene expression and splicing patterns towards that of differentiated neurons (as shown in **Fig 7e**), as well as the functional enrichment of the differentially spliced SRRM4 targets in GO terms such as “plasma membrane bounded cell projection organization” and “cell morphogenesis involved in neuron differentiation” (as shown in **Fig 4a**). To avoid inappropriately overstating the comparison with neuronal morphology, we have now modified several sections of the text as follows:

Results section title: *“SRRM4 expression induces neuronal-like expression, splicing patterns, and morphology in cancer cells”* to *“SRRM4 expression induces neuron-like expression and splicing patterns in cancer cells”*

Fig 7 legend: *“WT SRRM4-expressing cells develop numerous projections ~~indicative of neuronal differentiation~~ (orange arrows).”*

*“We also observed striking morphological changes in SH-SY5Y upon SRRM4 overexpression, where cells developed numerous ~~dendrite-like~~ membrane projections after 1 week of induction”*

*“...expression of SRRM4 in cancer cells leads to an upregulation of microexon inclusion, neuron-like expression and splicing patterns, and neuronal morphology...”* to *“...expression of SRRM4 in cancer cells leads to an upregulation of microexon inclusion and neuron-like expression and splicing patterns...”*

Minor comments

1)	Figures 1-7 legends should be updated to include a better description of the data presented (mean, medina, range, etc.) as well as statistical test and relevant p-values and symbols shown in the figures. Figure labels and graph axes should be made readable throughout the text, and axes should be labelled with better descriptions. For example Fig. 5E what exacly is "Max % inhibition (4days)?"

We have now ensured that all figure labels and axes are readable, and modified the axes in cases where they might have been unclear (Fig 2e, 2g, 3a, 7c). We have also included descriptions of statistical tests used in each figure, p-values and symbols, and descriptions of error bars wherever these were not included previously (legends of Fig 2a, 2b, 2e, 2g, 3b, 4a, 5e, 7a-f). Fig. 5e of the initial submission, referred to by the reviewer, has now been removed from the manuscript, as explained in response to major comment 6b.

2)	Fig. 2E shows increased fold change in SSRM4 expression in tumors vs. normal tissues, yet the remaining figures show decrease expression. The scale/legend should be probably be corrected.

We have now changed the title of the y-axis of **Fig 2e** from “Fold-change expression” to “Expression change (|T/N|)” to emphasize that the changes shown are the absolute values of the ratio of tumor/normal. We have also added a legend showing that points in blue are higher in tumors and red are lower in tumors.

3)	Fig. 2 - where does the list of 202 splicing factors comes from?

We now include the following description of the generation of the list of 202 splicing factors in the Methods section, under the heading *“Definition of splicing factor list*”:

*“The list of 202 splicing factors used in this study was derived from a published list of known splicing factors in mice [69] for which 1:1 orthologs could be identified in humans, plus 6 manual additions of human genes with evidence of splicing factor activity or family members thereof: ARGLU1 [70], PCBP2 [71], PRPF18 [72], SNIP1 [73], SRRM3 [22], and SFRS2 [74]. The complete list can be found in S1 Data.”*

For clarity, we also refer the reader to the methods section upon first mention of the list in the main text of the manuscript:

*“In fact, from a list of 202 known splicing factors (see Definition of splicing factor list in Methods), only SRRM4 had significantly decreased expression and increased promoter methylation across all tumor types (Fig 2d,f, S1-2 Data).”*

4)	Fig. 3A the dual scale is confusing; this should be plotted separately. What is the dark red color?

As recommended by the reviewer in major comment 3 above, we have modified **Fig 3a** to highlight separately microexons and non-microexons. The new figure legend clarifies the color scheme, where red exons are decreasing in tumors and blue are increasing, with the darker shade of each color representing microexons. We have moved the plot showing the distribution of ΔPSIs for all exons (previously shown in grey in **Fig 3a**) to a separate figure in the Supporting Information (**S2 Fig**).

5)	Fig. 3D - please clarify what this figure is comparing cancer cell lines vs. non transformed cell lines? If this is cancer cell lines vs. normal tissues, then this comparison is not acceptable as cell lines are known to be different from primary tissues and exhibit distinct gene expression and splicing profiles.

The reviewer is correct that the analysis previously shown in **Fig 3d** was a comparison of cancer cell lines with normal tissue data from VastDB. Our aim was to extend the findings of decreased microexon inclusion in tumors to more different cancer types than those available through TCGA. However, given the valid concern of the reviewer, we have removed this analysis from the revised manuscript. In its place, we have included a discussion of the findings of decreased microexon inclusion in glioblastoma reported by Reixachs-Sole et al 2020 (as described in major point 2 above).

6)	The authors state "The resulting list comprised between 468 exons in head and neck cancer to 1,884 in lung cancer, of which between 6.6% (head and neck cancer) and 12.7% (colorectal cancer) were micro-exons". Is the 12.7% in colorectal or lung? Is this a typo?

We apologize for the confusing wording of this sentence. While lung cancer has the greatest number of changing exons (1,884), colorectal cancer has the highest proportion of changing microexons (12.7%). To clarify this point, we have now added these summary statistics for each cancer type to Dataset S3 in a “summary” tab, and reworded the sentence in the text as follows:

*“The resulting list comprised between 468 and 1,884 exons depending on the tissue type, of which between 6.6% (head and neck cancer) and 12.7% (colorectal cancer) were microexons (Summary in S3 Data).”*

7)	The level of SRRM4 in vivo in tumor xenograft experiment should be shown in Fig 6.

As suggested, we now show the levels of SRRM4 induction in the tumors with doxycycline vs without doxycycline in Fig 6b and c (as insets), as measured by qPCR and quantified using the ΔΔCt method (described in the methods). Interestingly, we find that the SRRM4 induction level is much higher in the DM tumors (342-fold vs. uninduced) than the WT (22.3-fold vs. uninduced). Since the induction levels in the cell lines *in vitro* were similar between WT and DM cells (**S9 Fig**), we hypothesize that the difference observed *in vivo* is likely due to selective pressure for silencing of SRRM4 in the WT tumors, while there is no such selection in the DM tumors. Because these cell lines were generated using lentivirus, which randomly integrates into the genome, in a mixed population of cells some will have integrations in highly expressed loci and others in lowly expressed loci. It is therefore quite likely that over the course of several weeks of tumor growth, those with lower expression will come to dominate the population.

If the reviewer wishes, we can include a discussion of this result in the manuscript.

8)	Fig. 7C needs statistics and the results of the differential gene expression analysis should be provided as table.

As suggested, we have now added statistics to **Fig 7c** and updated the legend as follows:

*“Genes with increased expression in all 6 cell lines after SRRM4 induction (fold-change ≥ 2 compared to DM control). Bars represent the mean fold-change values, with error bars representing 95% CI. Red bars are genes with known neuronal function (GO: nervous system development) and/or neural-specific expression patterns. Fold-change values shown are significantly different from 0 (one-sample t-test, two-tailed, *p < 0.05, ** p < 0.01, *** p < 0.001, **** p < 0.0001)”*

We have also identified and corrected two other minor mistakes related to **Fig 7c**: 1) the y-axis label has been changed from “log2 FC (SRRM4 WT-DM)” to “log2 FC (SRRM4 WT/DM); and 2) in the legend of **Fig 7c**, “GO: neuron” has been changed to “GO: nervous system development”.

We have now included the differential gene expression analysis in the revision as **S7 Data**, and also updated figures 7a, b, c and f, and S6 data.

9)	Supplemental Fig. 6 is cited but it is not in the document.

We apologize for this oversight, as supplemental figs 5 and 6 were merged from an earlier version of the manuscript. We have now re-numbered the supplemental figures to include those added during the current revision.

10)	The discussion states "Notably, we find that prostate cancers and lung cancers taken as a whole follow the opposite trend as their neuroendocrine subtypes." - this is a potentially very exciting statement, but one would love to see that data in the paper.

We apologize for the poor wording of this sentence, which makes it sound as though the opposing findings in neuroendocrine tumors were our own findings. Our intention was to draw a contrast between our study and the published work of other groups who have reported an increase in SRRM4 expression in neuroendocrine subtypes of prostate and lung cancers, as cited in the same paragraph of the discussion. We have not analyzed these datasets ourselves because of the rarity of these tumors and their resulting lack of representation in TCGA. To avoid any confusion on this point, we have reworded this sentence as follows:

*“Notably, our findings suggest that prostate cancers and lung cancers on the whole follow the opposite trend as has been shown by others for their neuroendocrine subtypes [59,64].”*

---

Reviewer #3 (Steve Mount):

This paper presents evidence that SRRM4 silencing in tumors promotes growth and is associated with reduced inclusion of microexons. The two principal claims (first, that SRRM4 is silenced in tumors; second, that this decreased expression affects growth and induction of SRRM4 expression inhibits proliferation) are well supported.

The paper is well-written. I read it very carefully and have no significant suggestions for revision (which is extremely unusual for me).

The one suggestion I would make (not mandatory) is to consider logit(PSI) in order to identify those events whose altered splicing occurs in the context of nearly complete skipping or nearly complete inclusion (delta-logit(PSI) can be significant when delta-PSI is not).

We are grateful for the very positive comments of the reviewer, and for the suggestion to consider using logit(PSI) for the differential splicing analysis. We agree with the reviewer’s point that using an absolute ΔPSI cutoff has the disadvantage of filtering out significant changes in exons with close to 0 or 100% inclusion. We therefore repeated the analysis of the TCGA data using logit(PSI) to determine differentially spliced exons.

While this analysis did dramatically increase the detection of exons with near complete inclusion or exclusion in normal tissues (see **Fig 10a of this letter**, below), we observed that the signal was dominated by these exons, such that the exons with the highest Δlogit(PSI) values had extremely small ΔPSIs (**Fig 10b of this letter**). Because such small inclusion changes (e.g. 0.1% or 0.01%) are unlikely to have a physiologically relevant consequence, we believe that it would still be necessary to impose a ΔPSI cutoff to avoid this bias. Regardless, we note that the overall results using Δlogit(PSI) vs ΔPSI are very similar, with a majority of SRRM4 targets decreasing in cancer (see **Fig 10c of this letter**, compared with **Fig 3b** of the manuscript).

**Fig 10: Differential splicing analysis of TCGA data using logit(PSI).** A) Δlogit(PSI_Tumor-Normal_) is highest for events with PSI_Normal_ very close to 0 or 100. B) The vast majority of events with Δlogit(PSI) > 3 have ΔPSI < 1 (axes are split for clarity). C) Histogram of Δlogit(PSI_Tumor-Normal_) for all events (grey) vs. SRRM4 exons (blue/red). Only events with Δlogit(PSI) > 0.5 are shown.

---

ACADEMIC EDITOR COMMENTS:

1)	Referee 2, point 1: This is a key point. The SRRM4 levels indeed are very low and the authors need to provide convincing evidence that the observed differences between normal and tumor are not noise. The referee suggests several ways to do this: 1) compare SRRM4 changes to changes of other very low abundance transcripts, 2) demonstrate changes at the protein level and 3) provide more detailed statistical analysis to demonstrated significance. Showing only 3) would not be sufficient.

We understand the concern of the reviewer regarding the low levels of SRRM4 expression. We have responded to each of the three points highlighted by the editor individually below:

**Point 1**: As suggested by the reviewer, we have analyzed the changes in expression of lowly expressed splicing factor genes (all SFs with median RSEM < 5 for either tumor or normal group, excluding 0’s) compared to all splicing factor genes or SRRM4 alone. The results of this analysis are shown in **Fig 2 of this letter**. We found that the average tumor/normal ratio for lowly expressed genes was 1.1, which was comparable to that of all splicing factor genes (1.133) and not significantly different from 1 by one-sample t-test (p = 0.5911). In contrast, SRRM4 had an average ratio of 0.3142 across tissues, which is significantly different from 1 by one-sample t-test (p < 0.0001). Therefore, we conclude that there is no general bias towards decreased expression in tumors among lowly expressed splicing factors.

**Point 2:** Regarding this point, we are unable to demonstrate changes at the protein level in the TCGA data because the protein is not detected in any of the available proteomics datasets, and would require a targeted proteomics approach using tissue samples which we do not have available. However, the positive correlations between SRRM4 RNA expression and the PSIs of its target exons (shown in **S4a Fig**) support the assumption that SRRM4 RNA levels do change the amount of functional protein expressed and have an impact on the splicing patterns observed in cancer.

**Point 3**: We have provided a detailed description of the statistical analysis performed to detect differential expression in the Methods section under the heading “Statistical Information”:

*“Differential expression/methylation of splicing factors and differential exon inclusion were determined by two-tailed unpaired Wilcoxon-Mann-Whitney test, using the Benjamini Hochberg method to correct for multiple testing. The numbers of samples per group are indicated in S1 Table, and p- and q- values are reported in S1-3 Data.”*

As noted in our response to reviewer 2, we further provide the following evidence in support of the accuracy of our differential analysis:

1. It has been shown previously that RNAseq is sensitive enough to accurately quantify gene expression levels at values similar to those of SRRM4 in these samples (e.g. PMID: 24454679).
2. The datasets analyzed in this study contain large numbers of samples (n is in the range between 35 and 1135 with a mean of 384 samples per group; see **S1 Table**) resulting in a very high statistical power to detect differential expression between the tumor and normal groups.
3. We detect differential expression using a non-parametric Wilcoxon-Mann-Whitney test, which has been shown to have very low false positive rates on simulated expression data even under high noise level conditions (PMID: 12424116).
4. The coverage of TCGA samples ranges from 53 million to 140 million reads (with a mean of 79 million; only considering read pairs mapping to human genes). This is in fact in line with Illumina recommendations for RNAseq experiments ("Experiments looking for a more global view of gene expression, and some information on alternative splicing, typically require 30–60 million reads per sample"; source: https://support.illumina.com/bulletins/2017/04/considerations-for-rna-seq-read-length-and-coverage-.html) and are well beyond saturation levels of statistical power to detect differential expression given the high number of samples in each group (PMID: 24319002).
5. We see a consistent downregulation of SRRM4 across all tumor types tested (excluding liver, which has zero expression in normal tissue) (**Fig 2a**). We also note that if the change in SRRM4 expression was due to noise, there would be no implicit reason it should consistently change in the same direction across different tissue types, or within different samples in the same tissue type. This is supported by the fact that other lowly expressed splicing factors do not show the same consistency in the direction of change (**Fig 2** **of this letter**).

Taken together, we believe these points provide substantial evidence that we can confidently detect SRRM4 differential expression, and that these differences are not due to noise. In fact, consistently regulated “noise” is not noise but signal by definition, and our observation of strikingly consistent downregulation of SRRM4 in tumors, even at low levels, supports our hypothesis.

2)	Referee 2, point 4: This is another key point. There is the possibility that the observed phenotypes are due to effects that occur via SRRM4 but not via micro-exons. The authors should demonstrate and discuss the effect of SRRM4 on exons other than micro-exons and provide evidence that they do not contribute to phenotype, for example, by demonstrating that non-microexon events do not occur in cancers.

We apologize for the lack of clarity on this point. Reviewer 2 is correct that SRRM4 regulates exons that are not technically microexons. As shown by Irimia et. al. (PMID: 25525873), SRRM4-regulated exons have a strong tendency to be smaller than other alternative exons, which average ~140 nucleotides. While we define microexons here as exons ≤ 27 nucleotides in length, it is relevant to note that the exact definition of microexons vs. non-microexons depends on an arbitrary cutoff. By our definition, 61.3% of SRRM4 targets are microexons. Others have used a cutoff of 51 nucleotides (e.g. PMID: 25524026), by which definition 86.6% of SRRM4 targets would be considered microexons, while > 99% of SRRM4 targets are smaller than average (the distribution of exon lengths is shown in **Fig 5 of this letter**).

To assess the potential contributions of microexons vs longer SRRM4 target exons to the antiproliferative phenotype, as suggested by the editor, we analyzed separately the correlations of these exons PSIs with mitotic index (as shown combined in **Fig 4d** of the manuscript). We find that those with negative correlations are more likely to be microexons, while those with positive correlations are more likely to be non-microexons (**S5 Fig**). This finding, combined with the fact that a majority of SRRM4 targets are microexons in general, suggests that changes in microexon splicing contribute more to the antiproliferative phenotype than non-microexons. However, we cannot rule out that larger SRRM4 target exons contribute to the phenotype observed. We have summarized these results in the text as follows:

*“Furthermore, a majority of the SRRM4 target exons with significant negative MI correlations were ≤ 27 nt in length, while those with positive correlations tended to be longer (S5 Fig). This finding suggests that SRRM4 target microexons may contribute more to the antiproliferative phenotype than longer SRRM4 target exons; however, we cannot rule out the possibility that longer SRRM4 targets also contribute.”*

In addition, as suggested by reviewer 2, we now highlight microexons (≤ 27 nucleotides) in both **Fig 3a and c**. In both cases, it is clear that a majority of differentially spliced microexons have decreased inclusion in cancer. We have also included a new figure (**Fig 3d** of the revised manuscript) showing the percentages of differentially spliced microexons that decrease in each tumor type, which comprise a majority in each case and range from 60-87% depending on the tissue. Similarly, the GO enrichment analysis in **Fig 4a** relates to all SRRM4 targets, but repeating the analysis with only SRRM4 target microexons gives similar results (**Fig 6 of this letter**).

Thus, we conclude that the overall findings of our study hold when looking exclusively at microexons. However, because the majority of our study revolves around changes in SRRM4 expression (both as an observation in the TCGA data and in our experiments with cancer cell lines), we felt it more appropriate to report the findings on splicing changes of SRRM4 target exons taken as a whole.

3)	Physiological relevance: This is a difficult point to fully resolve, but I agree with Referee 1, point 4 that it would be reassuring to see that downregulation in non-malignant cells has an effect. The flipside of this is referee 2, point 6, highlighting that the overexpression experiments compare apples to oranges by introducing SRRM4 at much higher levels than physiologically observed, making its interpretation difficult. The suggested PCR analysis would give a better sense of how far off the comparison is.

We agree with the point of the editor and reviewers 1 and 2 regarding the physiological relevance of our experimental findings. We performed the qPCR analysis suggested by reviewer 2, and found that the maximum overexpression levels achieved in the cell lines with the highest concentration of doxycycline (~70- to 500-fold, **S9 Fig** in the revised manuscript) were indeed significantly larger than the changes observed in the tumors (~1.5- to 7-fold, **Fig 2e**). By using varying concentrations of doxycycline in the resazurin growth assays shown in **Fig 5a-c** and **S7 Fig**, we demonstrate the dose-dependence of SRRM4 expression on cell growth. However, as pointed out by reviewer 2, the phenotypic effects at the lowest dox concentrations are mild, making them difficult to quantify during the short time period of these growth assays.

We therefore performed a new experiment to demonstrate that the small differences we detect in SRRM4 expression can have an impact on cell growth over time. This experiment was designed to mimic the growth competition between cells with high and low levels of SRRM4, as would occur *in vivo* during tumor evolution. In this experiment, MDA-MB-231 cells with inducible expression of either WT or DM SRRM4 were mixed at a 1:1 ratio. The co-cultures were then passaged for several weeks in the presence of serial dilutions of doxycycline, such that the lowest concentration (31.3 ng/mL) achieved a similar level of SRRM4 induction as the change in SRRM4 expression observed between tumors and healthy tissues (2.6-fold, **S10a Fig** in the revised manuscript). Samples of the co-cultures were taken weekly, and the proportion of each cell line in the population was monitored by PCR using primers flanking the deleted region of SRRM4, allowing cells expressing WT or DM SRRM4 to be differentiated by the size of the PCR product. Compared to the ratio of WT to DM cells at day 1, we observed a dose- and time-dependent decrease in the WT/DM ratio that was significant after 2 weeks of co-culture at all concentrations tested (**Fig 5e-f** in the revised manuscript). We also performed the same experiment in HCT116 cells and observed similar but even stronger effects (**S11 Fig** in the revised manuscript), although the levels of SRRM4 induction were higher in this cell line with the same concentrations of doxycycline (**S10b Fig** in the revised manuscript). This result demonstrates that the effects of small changes in SRRM4 expression are amplified over time, and that the changes observed between tumors and normal tissues are sufficient to provide a growth advantage to tumor cells with lower SRRM4 expression.

We also attempted to downregulate SRRM4 in non-malignant cell lines, as suggested by reviewer 1, but we obtained contradictory results using three different shRNA sequences (one increased proliferation rate, one decreased proliferation rate, and one killed all the cells). We believe this is likely because the expression is very low and therefore the shRNA could be hitting off targets. However, we would contend that the assay described above demonstrates the same concept, as we use a cancer cell line with very low basal SRRM4 expression to express low levels of either WT SRRM4 (analogous to normal tissue) or an inactive mutant of SRRM4 (analogous to tumor tissue).
