# Supplementary material for: Silencing of SRRM4 suppresses microexon inclusion and promotes tumor growth across cancers
Source: PLoS Biol. 2021 Feb 23;19(2):e3001138. doi: 10.1371/journal.pbio.3001138 (PMC7935315; doi:10.1371/journal.pbio.3001138)
Supplement: Attachment — Submitted filename: Response to reviewer 20210120.docx [file pbio.3001138.s037.docx]

Rev. 2:

The authors have considerably revised and improved the manuscript.
A few points should still be clarified before publication.

Major comments
1) The author added a new experiment in Fig. 5e-f. The authors state they mixed WT or DM SRRM4 cells at a 1:1 ratio, yet at day 1 the ratio detected by PCR is not 1:1, raising the question of how quantitative and reproducible this assay is. Moreover, this experiment shows only one replicate and no control for the effects of doxycycline.

We understand the concerns regarding the new experiment shown in Fig 5e-f. The reviewer is correct that although the starting ratio of WT to DM cells was exactly 1:1, the PCR products were not produced in a 1:1 ratio. The issue seems to come from a PCR artifact resulting from the homology of the two products produced in the PCR reaction, leading to the formation of a chimeric product of intermediate size close to the WT band (See Fig 5f, reproduced below). This phenomenon has been reported by numerous other groups^[1]^, and unfortunately, our attempts to optimize the PCR reaction with different conditions and reagents failed to completely resolve the issue. For this reason, we normalized the ratio at each timepoint to the ratio at day 1 for each dox concentration, as shown in Fig 5e (reproduced below) and described in the figure legend (quoted below the figure).

*“Fig 5. E) Ratios of WT/DM SRRM4 in MDA-MB-231 co-cultures show a dose- and time-dependent decrease (normalized to day 1) that is significant at every doxycycline concentration tested (unpaired t-test using Holm-Sidak method to correct for multiple comparisons; * p < 0.05, ** p < 0.01, *** p < 0.001). Results shown represent the mean of three independent experiments, and error bars represent standard deviation. F) Representative PCR showing the decreased WT/DM ratios of MDA-MB-231 co-cultures over time. WT/DM ratios quantified by densitometry are shown below each sample.”*

We apologize if we did not explain it well, but as stated in the legend of Fig 5, the experiment was repeated three times, and the graph shown in Fig 5e shows the mean of the three replicates with the standard deviation. As evidenced by the small error, the assay is highly reproducible, and the change in WT/DM ratio is statistically significant. As for its quantitative nature, due to the aforementioned artifact we cannot correlate the band intensity with the percentage of each population in a precise manner, but is quite obvious that SRRM4 DM outcompetes the SRRM4 WT population over time, which was the objective of this experiment.

Finally, as far as the control for the effects of doxycycline, we can see from the experiments shown in 5a and c that doxycycline concentrations up to 1000 ng/mL have no effect on the growth rate of the parental cell line MDA-MB-231 or the DM SRRM4-expressing cells. Therefore, we conclude that the effects of doxycycline on cell growth are exclusively due to the dox-induced expression of WT-SRRM4, particularly at the low concentrations used in the competition assay.

Finally, the interpretation of this growth competition assay in Fig.5, as well as the xenograft assays in Fig.6, is challenging because the DM cell line grows more slowly, even without DOX (induced), compared to the WT cell line. As show in Fig. 6, uninduced WT and DM cells reach a tumor volume of a 100 at 20 and 40 days respectively.

We respectfully disagree with the reviewer’s interpretation that the DM cell line grows more slowly than the WT. If this was the case, then the ratio of WT/DM should increase over time, but our results in Fig 5e-f show the opposite, even at the lowest concentration of dox tested. This means the WT cells are growing slower than DM, and the magnitude of this effect is dependent on the levels of SRRM4 induced.

Regarding Fig 6, the reviewer is correct that in this experiment the DM tumors were induced with dox at a later timepoint post-implantation than the WT. However, we would like to clarify several points that suggest this is not in fact due to a difference in pre-induction growth rate of the tumors. First, in a pilot xenograft experiment with the same cell lines, this difference in tumor growth rate pre-induction was not observed (Fig 1 of this letter). Second, a side-by-side comparison of the unnormalized growth curves from the full-scale xenograft experiment in Fig 6 shows that the DM tumors were significantly smaller than the WT at the first measured timepoint post-implantation (6.6 mm^3^ vs 18.4 mm^3^ at day 7, Fig 2a of this letter), suggesting that there may have been fewer DM cells implanted to begin with. However, once the DM tumors reached the same size as the WT, the pre-induction growth rates were identical (Fig 2b of this letter). In addition, the DM tumors were significantly larger than the WT tumors at the time of induction (50.6 mm^3^ vs. 68.0 mm^3^), which is not represented in Fig 6 because the tumor volumes are normalized to the volume at the time of induction.

Taken together, these pieces of evidence suggest that there is no obvious difference in proliferation rate between uninduced WT and DM tumors. Regardless, the reported finding that induction of SRRM4 expression with doxycycline had no effect on the growth of the DM tumors is unaffected.

**Fig 1. Growth of WT and DM SRRM4 tumors in a pilot xenograft experiment shows no significant difference in pre-induction growth rate.** n = 12 tumors per group; error bars represent standard deviation.

**Fig 2. Growth of WT and DM SRRM4 tumors in the full-scale xenograft experiment shows difference in implantation volume, but not in pre-induction growth rate.** A) Side-by-side comparison of the WT and DM tumor growth over time. WT tumors were larger at the first measured timepoint than DM tumors. B) DM growth curve transposed to overlay WT growth curve (-8 days) shows identical pre-induction growth rates. DM tumors were larger at the time of induction than WT tumors. n = 12 tumors per group; error bars represent standard deviation.

Therefore experiments in Fig. 5e-f should either be removed, or repeated with appropriate controls. The current version shows the growth difference between the two clones, and does not point out necessarily to an effect of SRRM4.

Because the experiments shown in Fig 5e-f were done to address the concerns raised during the first round of revisions, we did not believe it would be appropriate to remove them from the manuscript. Furthermore, as detailed above, the control for the effects of doxycycline on cell growth are shown in Fig 5a and c, and we are not aware of another method by which we could control for these effects. Therefore, we hope the reviewer will be satisfied with the clarifications we have provided and approve of leaving the experiment in the final version of the manuscript. However, if these explanations are not satisfactory, we agree to remove the experiment from the manuscript.

2) In Fig. 7 from their response, the authors show that indeed the level of expression of SRRM4 WT and SRRM4 DM are different at the same doxycycline concentration. Therefore they should acknowledge in the text that the lack of effect of the DM SRRM4 on cell proliferation could be due to lower expression.

We understand the concern that differences in expression levels of WT and DM SRRM4 could underlie differences in proliferation. However, as shown in Fig 3 of this letter below, growth inhibition by WT SRRM4 is greater with lower concentrations of dox (green arrows) than at the highest concentration of DM SRRM4 (pink arrow), despite having lower levels of expression. Furthermore, quantification of SRRM4 in the other 5 cancer cell lines by Western blot (S8 Fig) and qPCR (S9 Fig) shows the expression of WT SRRM4 is slightly higher in some cell lines and slightly lower in others, whereas the proliferation inhibition by only WT SRRM4 is completely consistent in all cell lines (S7 Fig). Taken together, these results demonstrate that the lack of effect of DM SRRM4 is not due to lower expression.

**Fig 3:** **WT SRRM4 inhibits proliferation at lower levels of protein induction than the highest level of DM SRRM4.** The top panel shows proliferation assay results from Fig 5B-C, and the bottom panel shows protein level quantification by densitometry.

Minor comments
1) As part of best reproducibility practices, please add versions of computation tools, links to github pages if available, and specific parameters used.

We have now added the version number, specific parameters, and link to the github page of vast-tools to the methods section as follows:

*“To determine differential microexon splicing, we analyzed raw RNA-seq files using VAST-TOOLS (v2.0.2), a computational pipeline designed specifically for the quantification of microexon inclusion levels [1] (https://github.com/vastgroup/vast-tools). We ran VAST-TOOLS on 6908 samples (S1 Table). Using VAST-TOOLS align we first mapped the reads to the human reference genome hg38 and to quantify PSI levels for each exon and sample (“vast-tools align” with species --Hs2 and default parameters, plus --noIR and --EEJ counts options). We used VAST-TOOLS tidy function to remove exon quantifications with very low quality (“vast-tools tidy” with default parameters, plus --noVLOW option).”*

2) The word 'hypersilenced' should be replaced by 'silenced' throughout the text and title.

We have now replaced “hypersilenced” by “silenced” throughout the text and title as suggested.

3) The title should be edited to reflect more accurately the findings described in the paper. What is the meaning of basal micro-exons in the title?

By referring to “basal” microexon inclusion we meant to convey the normally low levels of inclusion in non-neural tissues. However, we understand that this term might generate confusion, and have now removed it from the title and text. We believe the title now more accurately reflects the findings described in the paper.

1. References:

   Shuldiner, A. R.; Nirula, A.; Roth, J. Hybrid DNA Artifact from PCR of Closely Related Target Sequences. *Nucleic Acids Res* **1989**, *17* (11), 4409.

   Brakenhoff, R. H.; Schoenmakers, J. G.; Lubsen, N. H. Chimeric CDNA Clones: A Novel PCR Artifact. *Nucleic Acids Res* **1991**, *19* (8), 1949.

   Wang, G. C.; Wang, Y. The Frequency of Chimeric Molecules as a Consequence of PCR Co-Amplification of 16S RRNA Genes from Different Bacterial Species. *Microbiology (Reading)* **1996**, *142 ( Pt 5)*, 1107–1114.

   Kanagawa, T. Bias and Artifacts in Multitemplate Polymerase Chain Reactions (PCR). *Journal of Bioscience and Bioengineering* **2003**, *96* (4), 317–323.

   Kalle, E.; Kubista, M.; Rensing, C. Multi-Template Polymerase Chain Reaction. *Biomol Detect Quantif* **2014**, *2*, 11–29.
